# Supplementary material for: Forward design of a complex enzyme cascade reaction
Source: Nat Commun. 2016 Sep 28;7:12971. doi: 10.1038/ncomms12971 (PMC5052792; doi:10.1038/ncomms12971)
Supplement: Supplementary Information — Supplementary Figures 1-13, Supplementary Tables 1-24, Supplementary Note 1, Supplementary Methods and Supplementary References. [file ncomms12971-s1.pdf]

Supplementary Figures

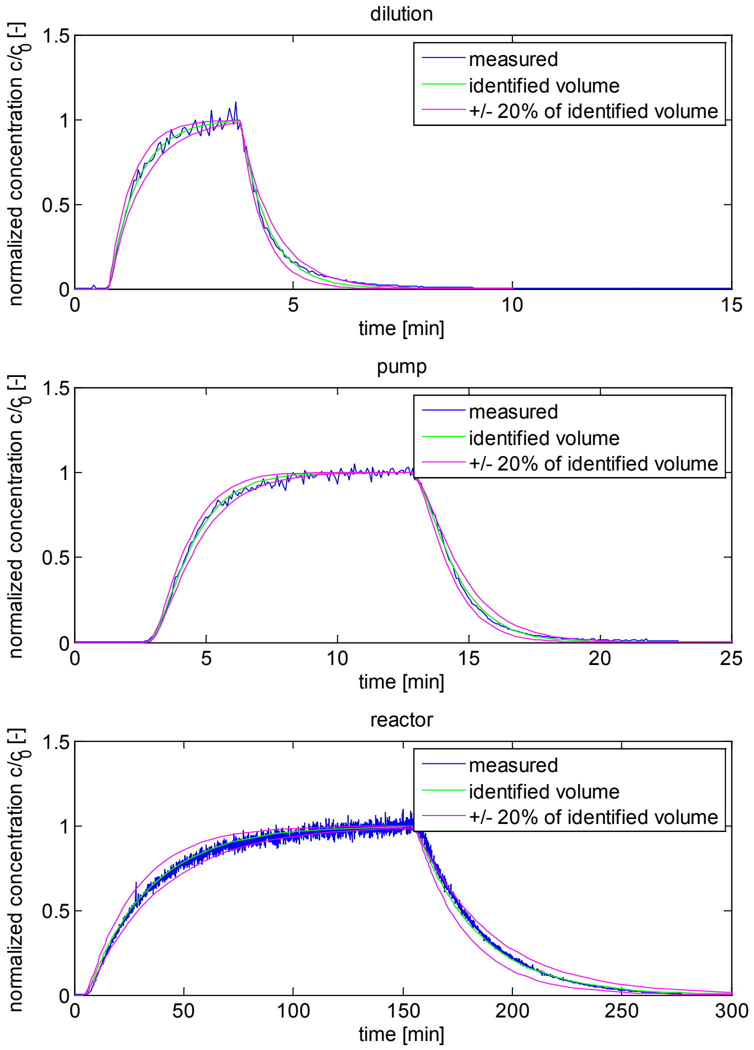

Supplementary Figure 1: Sensitivity of the model performance to errors in identified system volumes. Experimental and simulation results for three different identified volumes (dilution element, pump, and reactor) and for hypothetical changes in these volumes of +/- 20%.

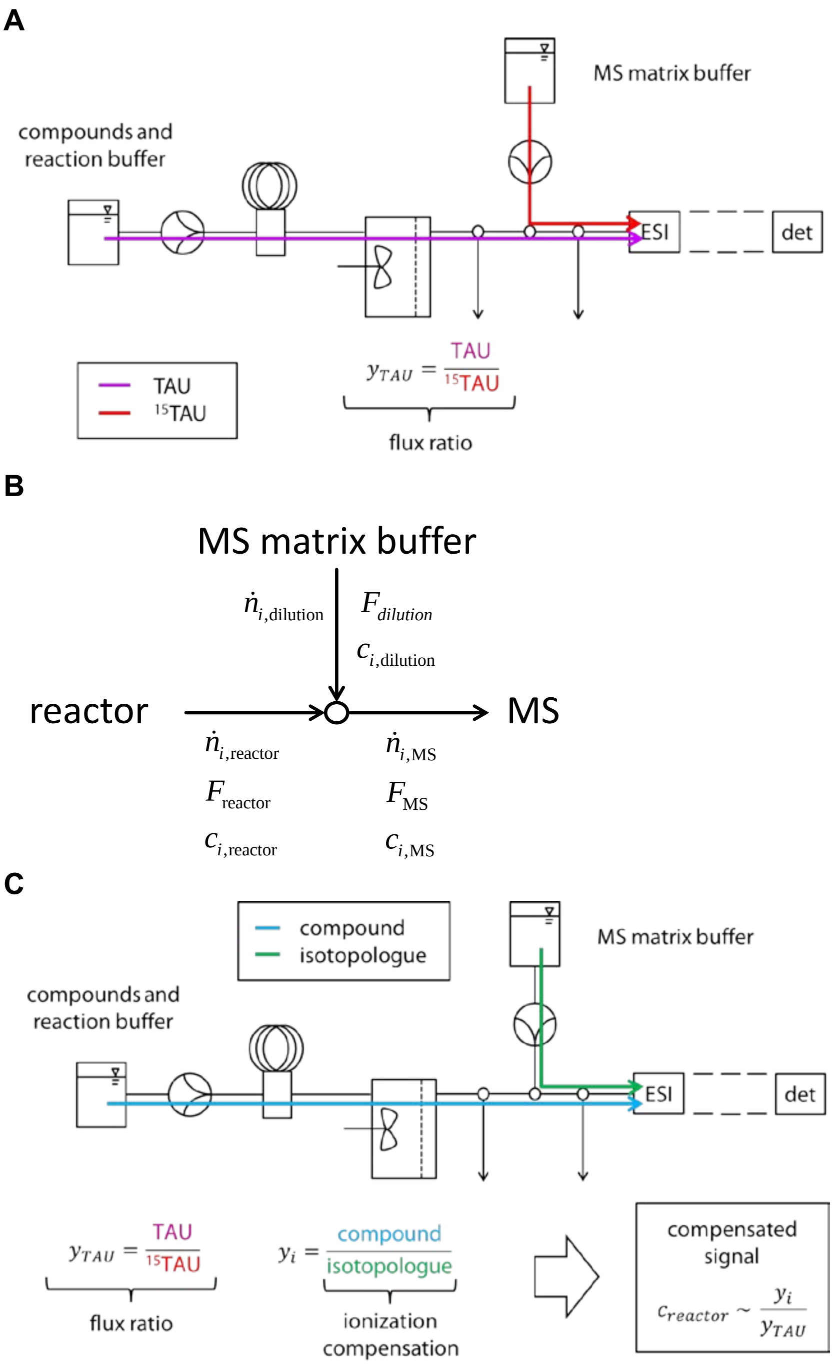
A
MS matrix buffer
compounds and reaction buffer
ESI
det
TAU
15TAU
$y_{TAU} = \frac{TAU}{^{15}TAU}$
flux ratio
B
MS matrix buffer
$\dot{n}_{i,\text{dilution}}$
$F_{dilution}$
$c_{i,\text{dilution}}$
reactor
MS
$\dot{n}_{i,\text{reactor}}$
F_{reactor}
$c_{i,\text{reactor}}$
$\dot{n}_{i,\text{MS}}$
F_{MS}
$c_{i,\text{MS}}$
C
compound
isotopologue
MS matrix buffer
compounds and reaction buffer
ESI
det
$y_{TAU} = \frac{TAU}{^{15}TAU}$
flux ratio
$y_i = \frac{\text{compound}}{\text{isotopologue}}$
ionization compensation
compensated signal
$c_{reactor} \sim \frac{y_i}{y_{TAU}}$

D

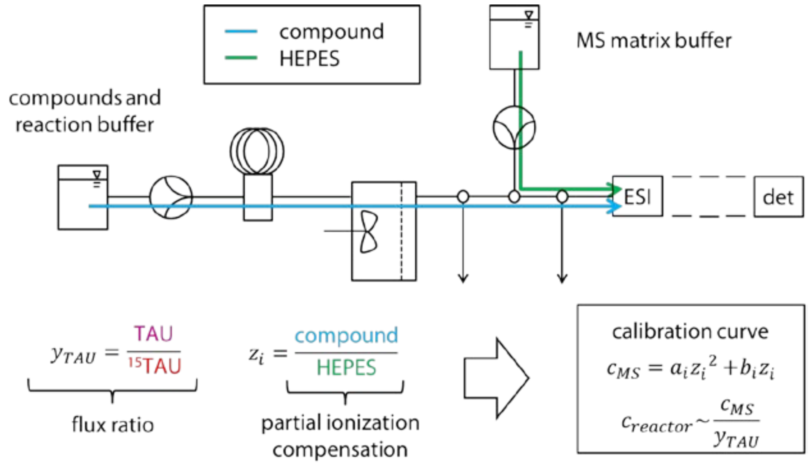

Supplementary Figure 2: Illustrations of the metabolite quantification methods and the flux monitoring. A: Known concentrations of TAU and ^{15}TAU were added to the reaction and the MS matrix buffer and allowed the calculation of the flux ratio of reactor effluent and matrix buffer. **B**: Fluxes at the first mixing tee after the reactor. Reactor effluent is diluted with MS matrix buffer containing different compounds essential for calibration. Abbreviations: $\dot{n}$ molar flux, c concentration, F flow rate. **C:** During experiments, unlabeled compounds came from the reactor and isotopologues were added at a known concentration to the MS matrix buffer. The relation between the compounds could be used to quantify the reactor concentration if the flux distribution (see A) was known. **D:** HEPES was added as a standard to the MS matrix buffer. Recorded calibration curves and the flux ratio information from the TAU system (see A) also allowed the quantification of the reactor concentration.

A

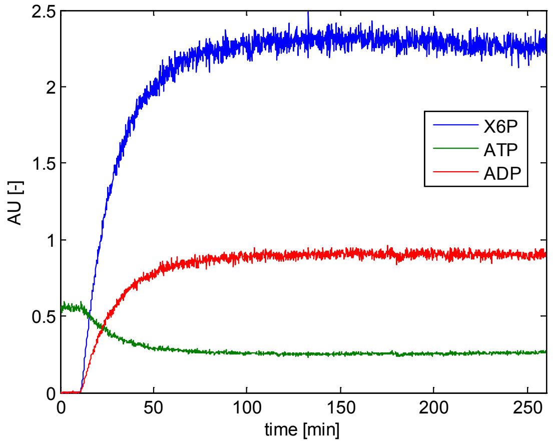

B

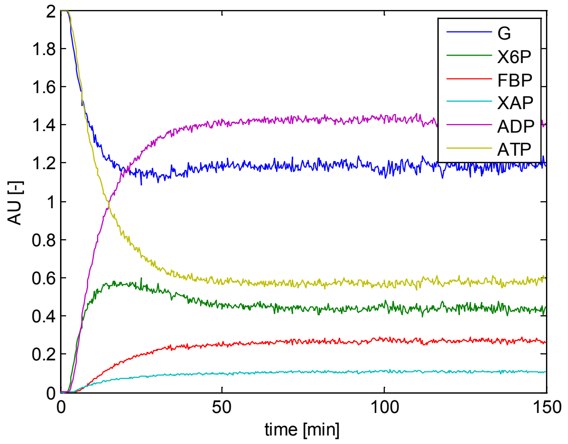

C

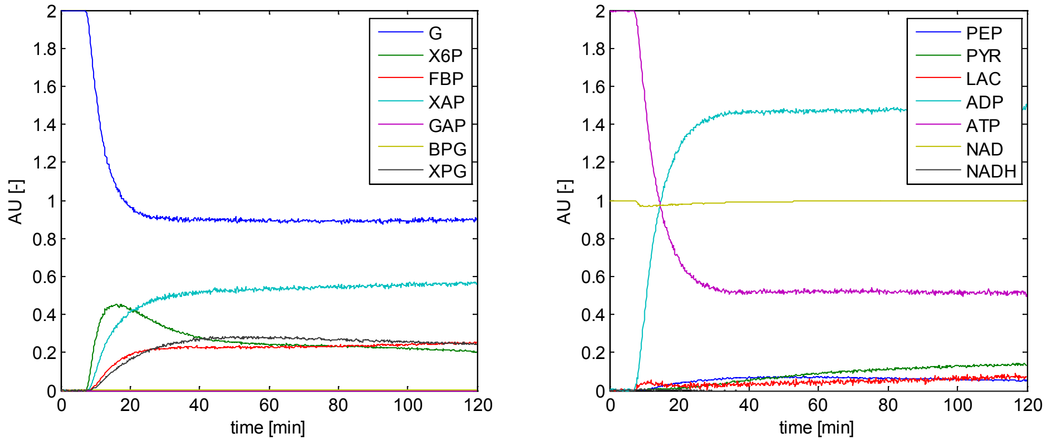

Supplementary Figure 3: Enzyme stability. A: Glk. After initial enzyme injection steady-state concentrations of substrate and pr oducts remain constant for 4 hours. **B:** Upper glycolysis. After initial enzyme injection steady-state concentrations remain constant for 150 min. **C:** Full glycolysis. After initial enzyme injection steady-state concentrations of substrates, intermediates and products remained constant for 120 min.

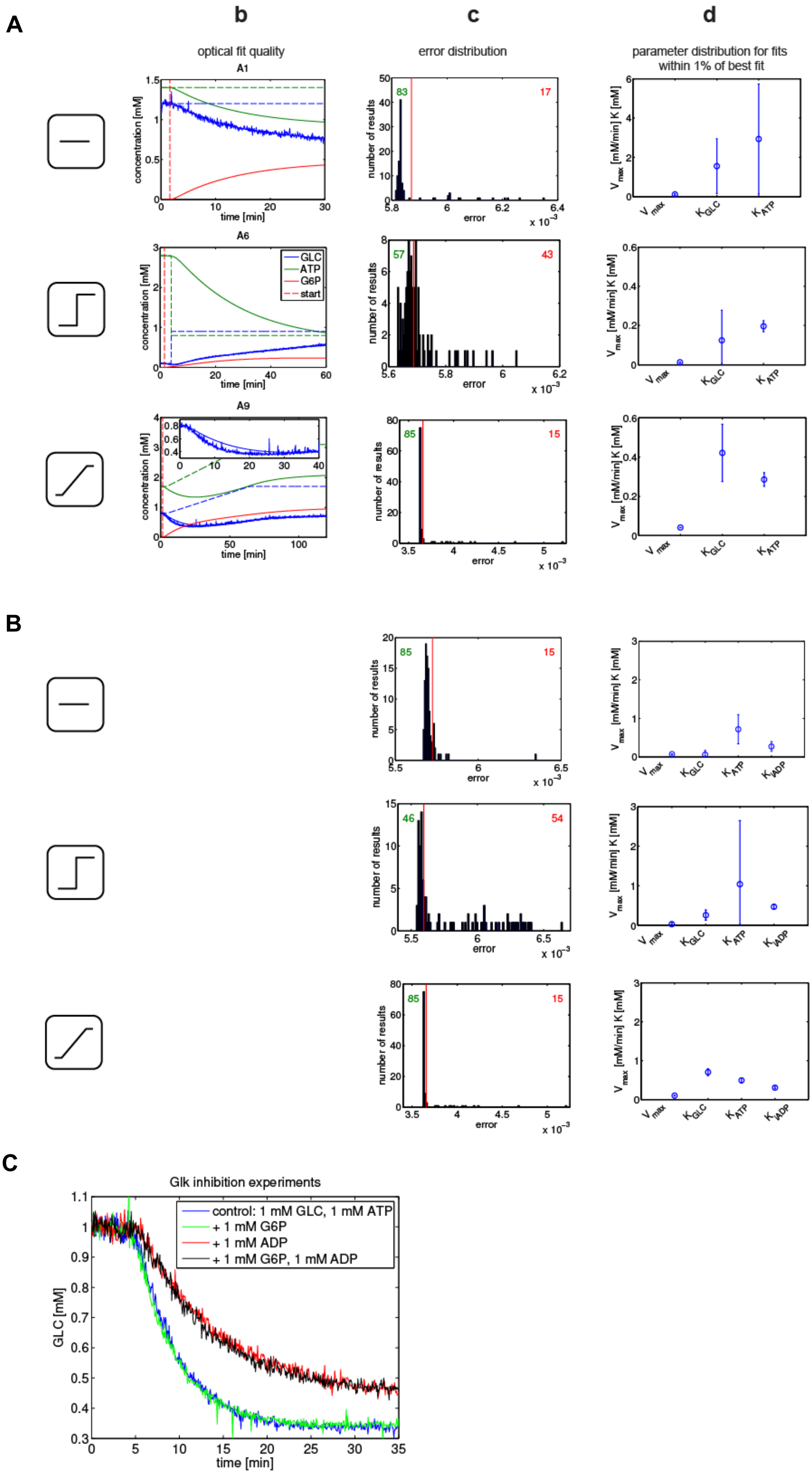
A
B
C
b
c
d
optical fit quality
error distribution
parameter distribution for fits within 1% of best fit
A1
A6
A9
concentration [mM]
time [min]
number of results
error
GLC
ATP
G6P
start
83
17
57
43
85
15
85
15
46
54
85
15
Glk inhibition experiments
control: 1 mM GLC, 1 mM ATP
+ 1 mM G6P
+ 1 mM ADP
+ 1 mM G6P, 1 mM ADP
GLC [mM]
time [min]

Supplementary Figure 4: Correction of the structure of the rate equation of Glk. A, B: Analysis of parameter estimations based on rate equation without (A) or with (B) inhibition by ADP. Each row displays the results for one type of input function. Column [a]: Type of input function applied. [b]: Measured concentration time series and s imulation with the best identified parameter set. The start of reaction by enzyme injection is indicated by the stippled red line. The only observed concentration is GLC (blue line), the concentration of other compounds is a result of the simulation. Stippled lines in blue or green indicate the simulated feed profile for GLC (blue) or ATP (green). Column [c] Error distribution achieved by the optimizer in 100 runs. The number in green at the left of each picture indicates the number of runs with an error which was not more than 1% larger than the smallest achieved error. The 1% criterion is indicated by the red line. The number in red at the right of each picture indicates the number of runs with an er ror more than 1% larger. Column [d]: Mean and standard deviations of the parameters that led to a qual ity of fit within 1% of the minimal error. **C:** Experimental confirmation for an inhibition of Glk by ADP. Recorded concentration time series for GLC in the presence or absence of ADP or G6P as possible inhibitors already in the feed. Mentioned concentrations refer to the initial compound concentrations in the reactor at the time point at which the experiment was started (addition of 1 U of Glk after 3 min) and to the concentrations in the feed. Note that the addition of only G6P does not produce a difference in the concentration time series as compared to the control, suggesting that G6P is not an inhibitor.

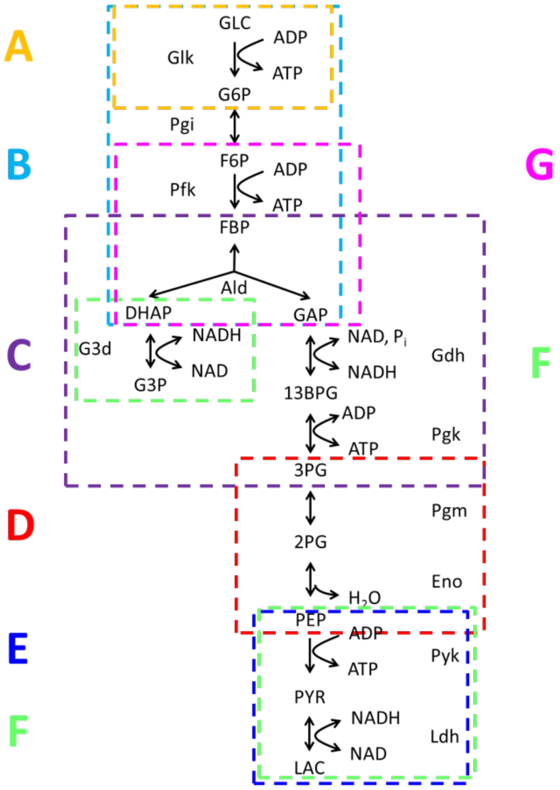

Supplementary Figure 5: Subsystems of the parameter estimation procedure.

A

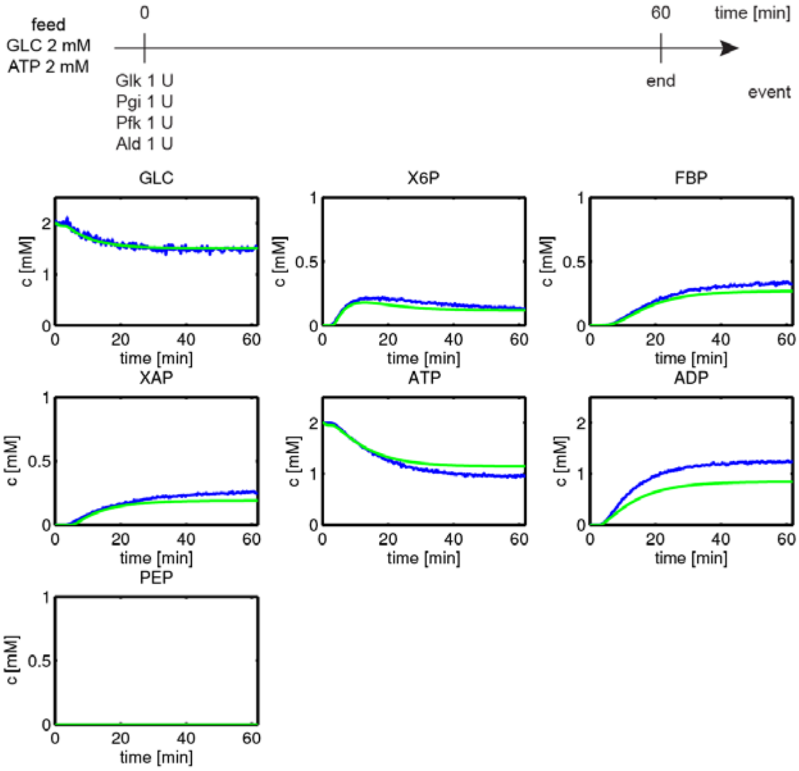

feed
GLC 2 mM
ATP 2 mM
0
60
time [min]
Glk 1 U
Pgi 1 U
Pfk 1 U
Ald 1 U
end
event
GLC
X6P
FBP
XAP
ATP
ADP
PEP
c [mM]
time [min]

B

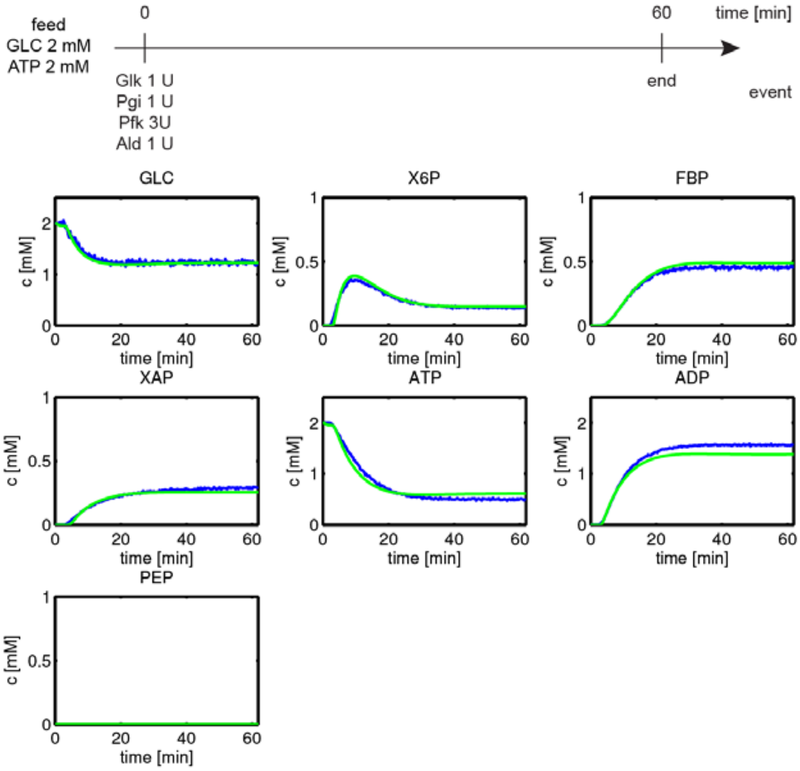

feed
GLC 2 mM
ATP 2 mM
0
60
time [min]
Glk 1 U
Pgi 1 U
Pfk 3U
Ald 1 U
end
event
GLC
X6P
FBP
XAP
ATP
ADP
PEP
c [mM]
time [min]

C

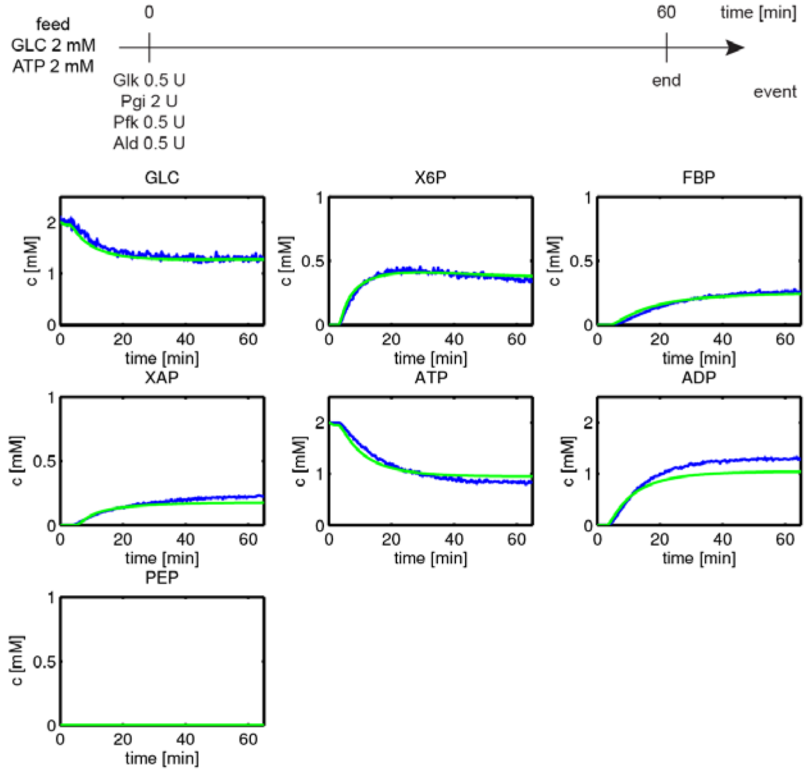
feed
GLC 2 mM
ATP 2 mM
0
60
time [min]
Glk 0.5 U
Pgi 2 U
Pfk 0.5 U
Ald 0.5 U
end
event
GLC
X6P
FBP
XAP
ATP
ADP
PEP
c [mM]
time [min]

D

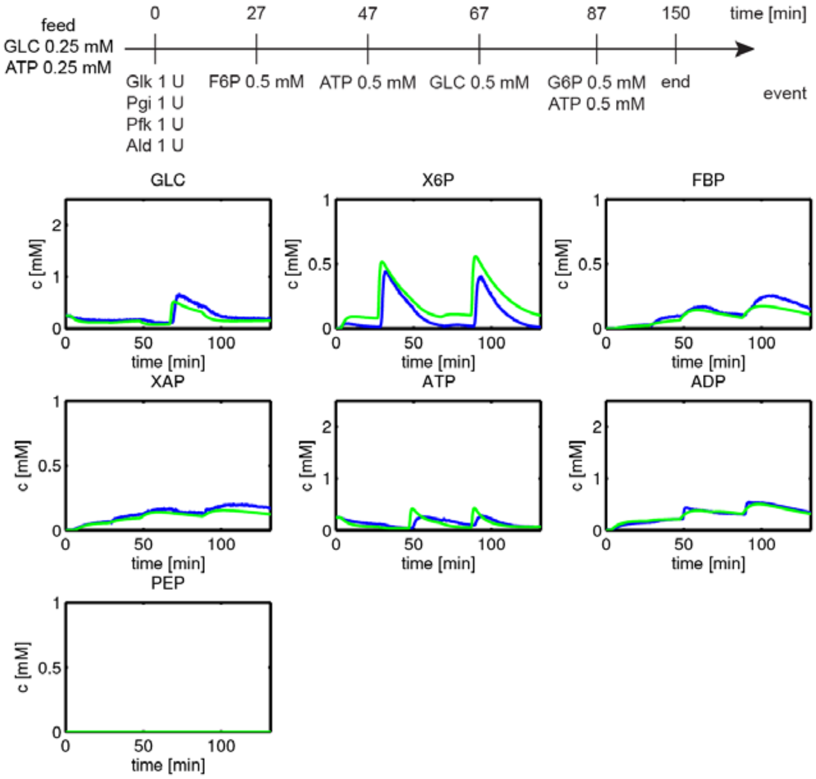
feed
GLC 0.25 mM
ATP 0.25 mM
0
27
47
67
87
150
time [min]
Glk 1 U
Pgi 1 U
Pfk 1 U
Ald 1 U
F6P 0.5 mM
ATP 0.5 mM
GLC 0.5 mM
G6P 0.5 mM
ATP 0.5 mM
end
event
GLC
X6P
FBP
XAP
ATP
ADP
PEP
c [mM]
time [min]

E

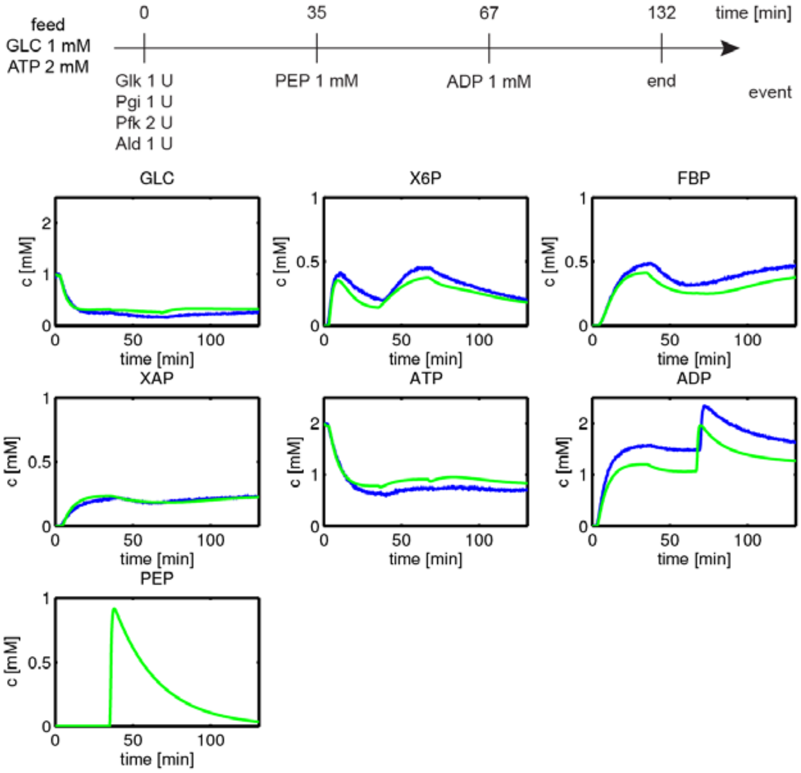

F

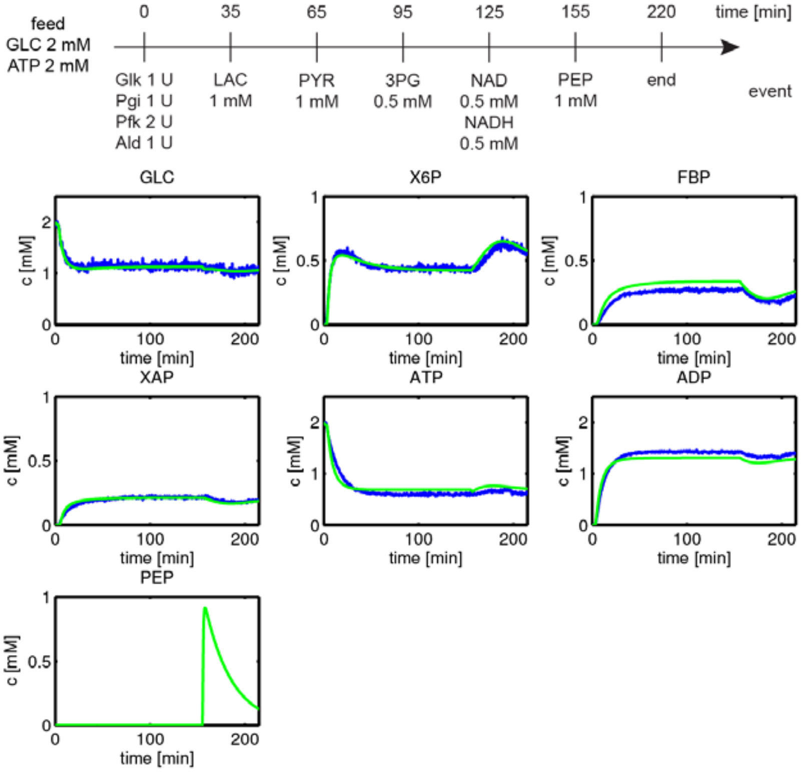

Supplementary Figure 6: Experiments for the parameter estimation of subsystem B, experiment and fit. Each section of the figure represents one of the experiments B1 to B6 and features a summary of the experiment in the upper panel (see also Supplementary Table 6), followed by the concentration time series (measured: blue, best-fit simulation: green). The

parameters for the best-fit simulation were taken from Supplementary Table 7. **A:** B1; **B:** B2; **C:** B3; **D:** B4; **E:** B5; **F:** B6.

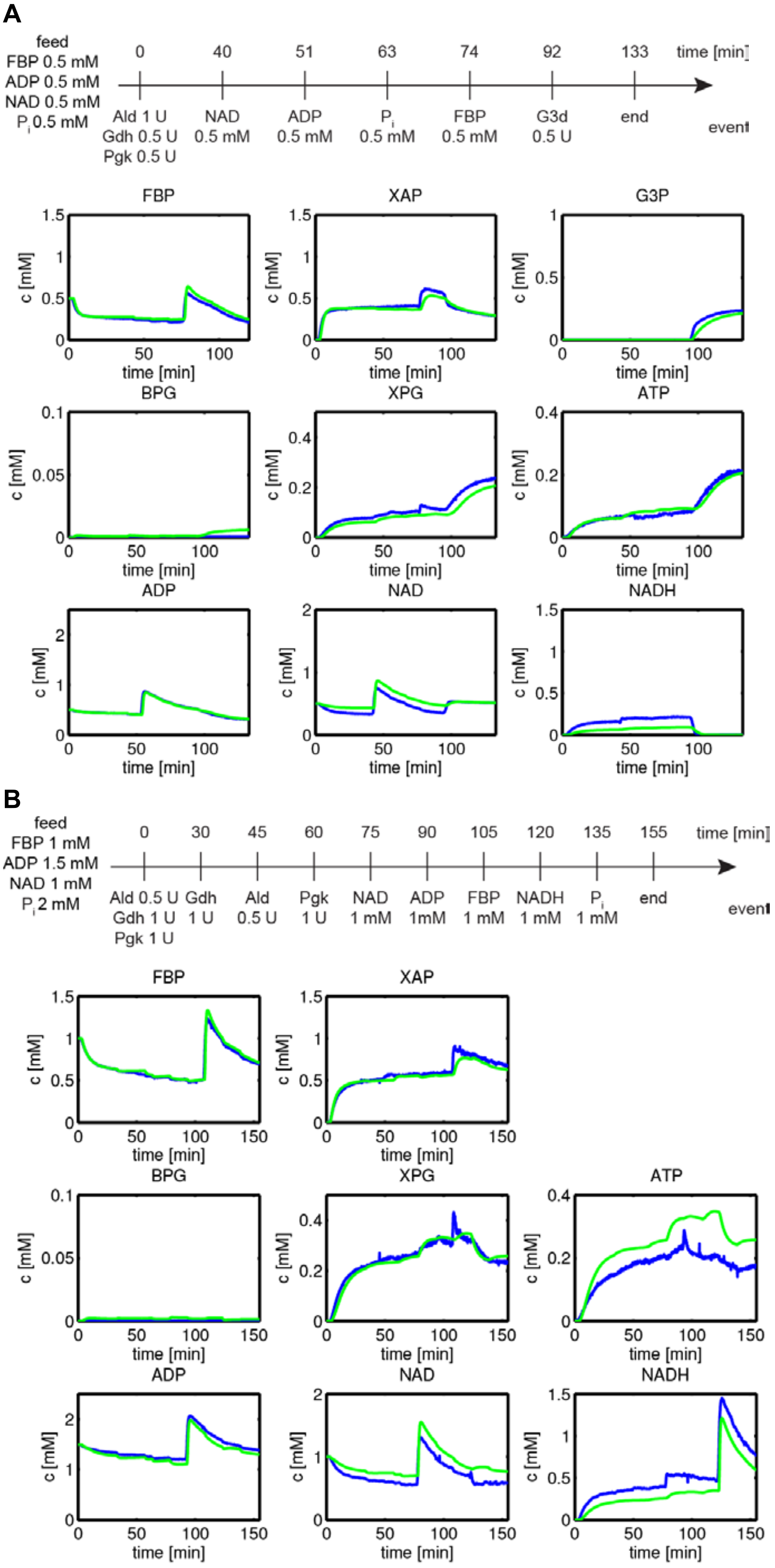
A
feed
FBP 0.5 mM
ADP 0.5 mM
NAD 0.5 mM
P_i 0.5 mM
0 40 51 63 74 92 133 time [min]
Ald 1 U Gdh 0.5 U Pgk 0.5 U
NAD 0.5 mM
ADP 0.5 mM
P_i 0.5 mM
FBP 0.5 mM
G3d 0.5 U
end
event
FBP
XAP
G3P
BPG
XPG
ATP
ADP
NAD
NADH
c [mM]
time [min]
B
feed
FBP 1 mM
ADP 1.5 mM
NAD 1 mM
P_i 2 mM
0 30 45 60 75 90 105 120 135 155 time [min]
Ald 0.5 U Gdh 1 U Pgk 1 U
Gdh 1 U
Ald 0.5 U
Pgk 1 U
NAD 1 mM
ADP 1mM
FBP 1 mM
NADH 1 mM
P_i 1 mM
end
event
FBP
XAP
BPG
XPG
ATP
ADP
NAD
NADH
c [mM]
time [min]

C

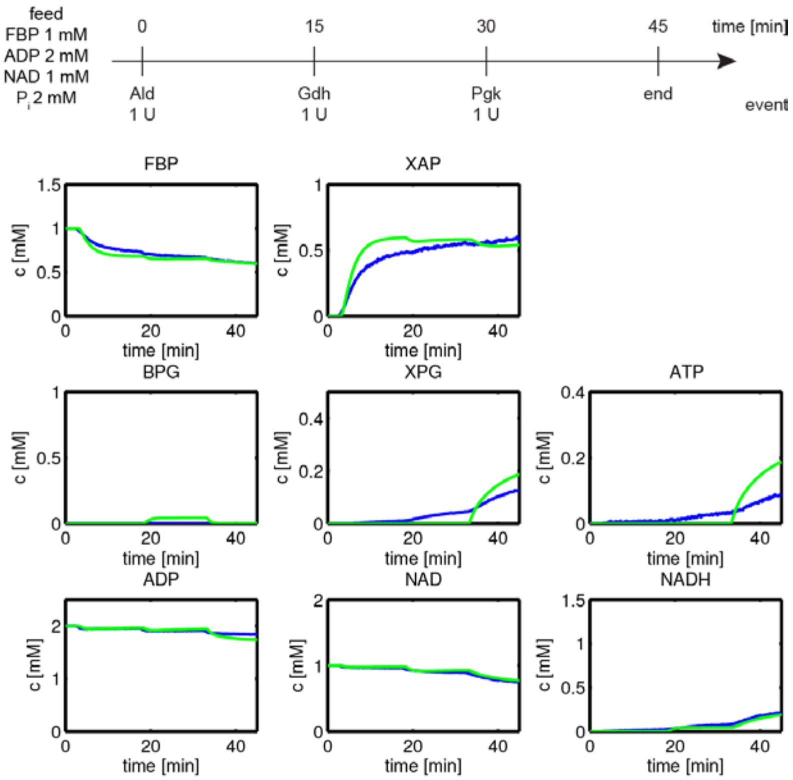

D

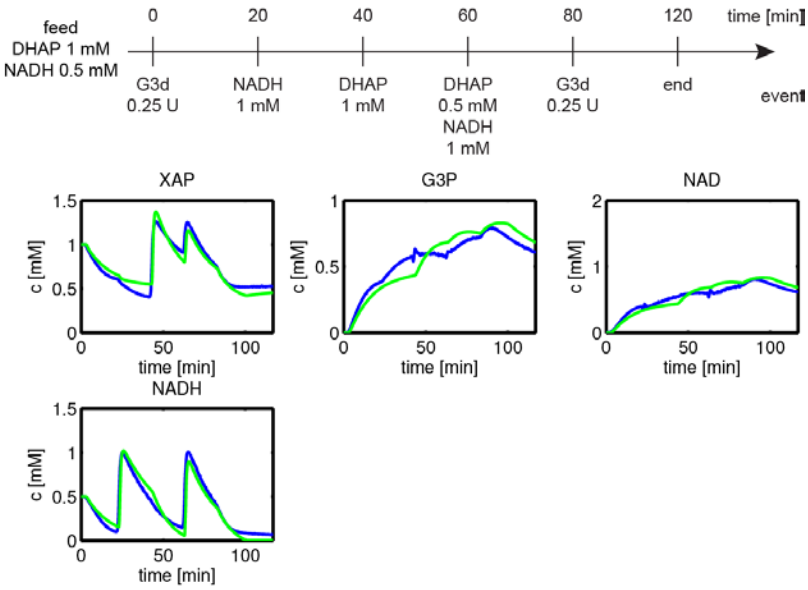

Supplementary Figure 7: Experiments for the parameter estimation of subsystem C, experiment and fit. Each section of the figure represents one of the experiments C1 to C4 and features a summary of the experiment in the upper panel (see also Supplementary Table 8), followed by the concentration time series (measured: blue, best-fit simulation: green). The parameters for the best-fit simulation were obtained from Supplementary Table 9. **A:** C1; **B:** C2; **C:** C3; **D:** C4. Note that for experiment C3, simulation with the parameter set of Supplementary Table 9 showed a lower fit quality than the other experiments. This can be explained by impurities of the enzymes as can be seen for the injection of 1 U of Gdh after 15 min: theoretically only BPG should be formed but instead one can see an increase in both

XPG and A TP, both products of Pgk. This indicates that the applied preparation of the enzyme Gdh might have been contaminated with Pgk.

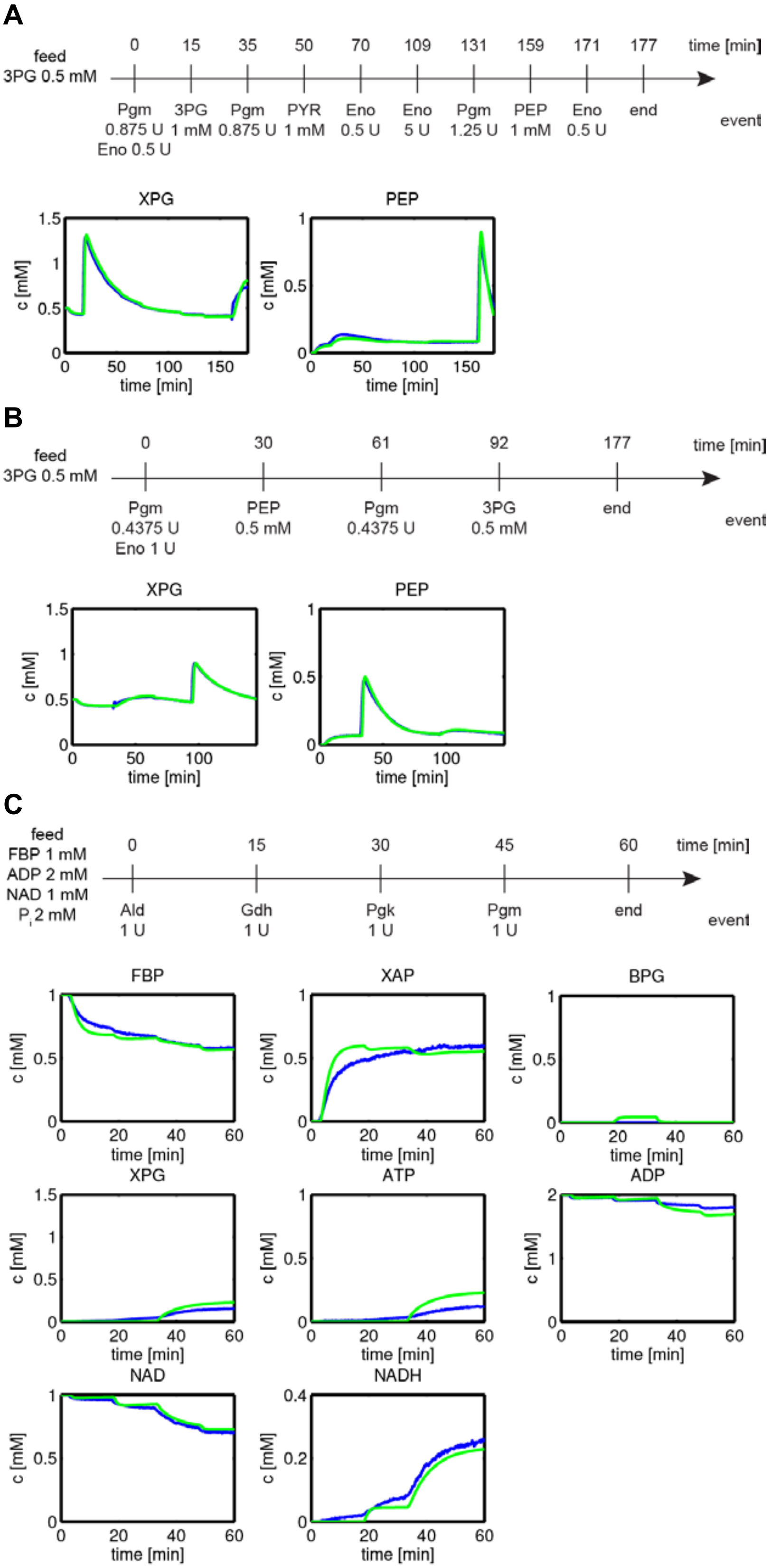

Supplementary Figure 8: Experiments for the parameter estimation of subsystem D, experiment and fit. Each section of the figure represents one of the experiments D1 to D3 and features a summary of the experiment in the upper panel (see also Supplementary Table 10), followed by the concentration time series (measured: blue, best-fit simulation: green).

The parameters for the best-fit simulation were obtained from Supplementary Table 11. **A:** D1; **B:** D2; **C:** D3.

A

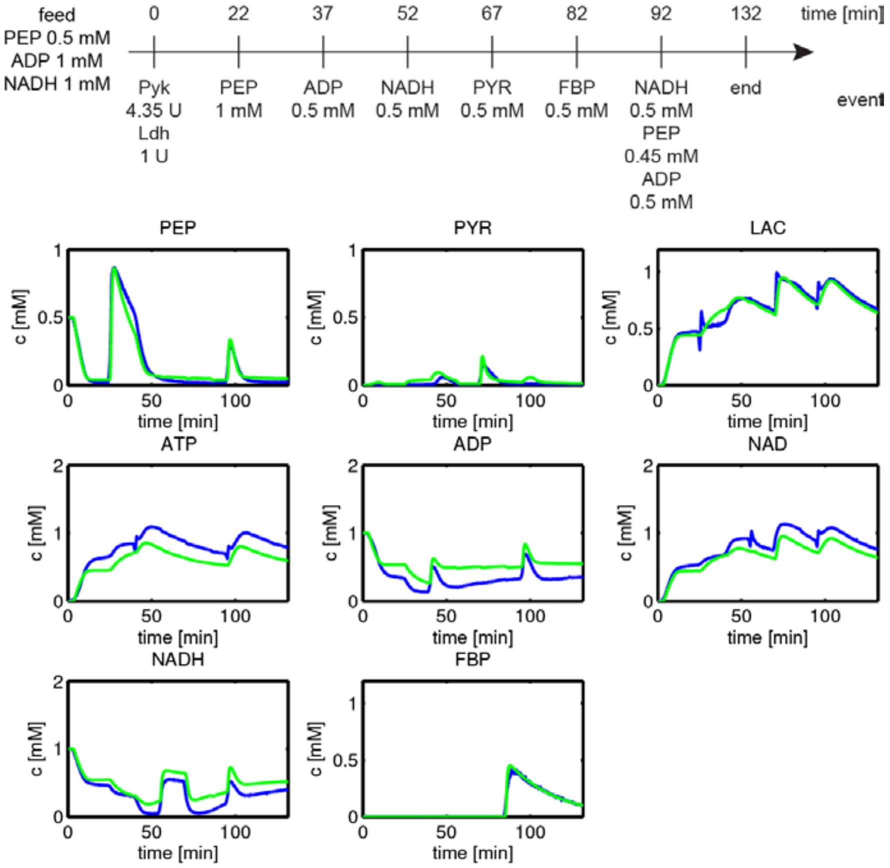
feed
PEP 0.5 mM
ADP 1 mM
NADH 1 mM
0
22
37
52
67
82
92
132
time [min]
Pyk 4.35 U Ldh 1 U
PEP 1 mM
ADP 0.5 mM
NADH 0.5 mM
PYR 0.5 mM
FBP 0.5 mM
NADH 0.5 mM PEP 0.45 mM ADP 0.5 mM
end
event
PEP
PYR
LAC
ATP
ADP
NAD
NADH
FBP
c [mM]
time [min]

B

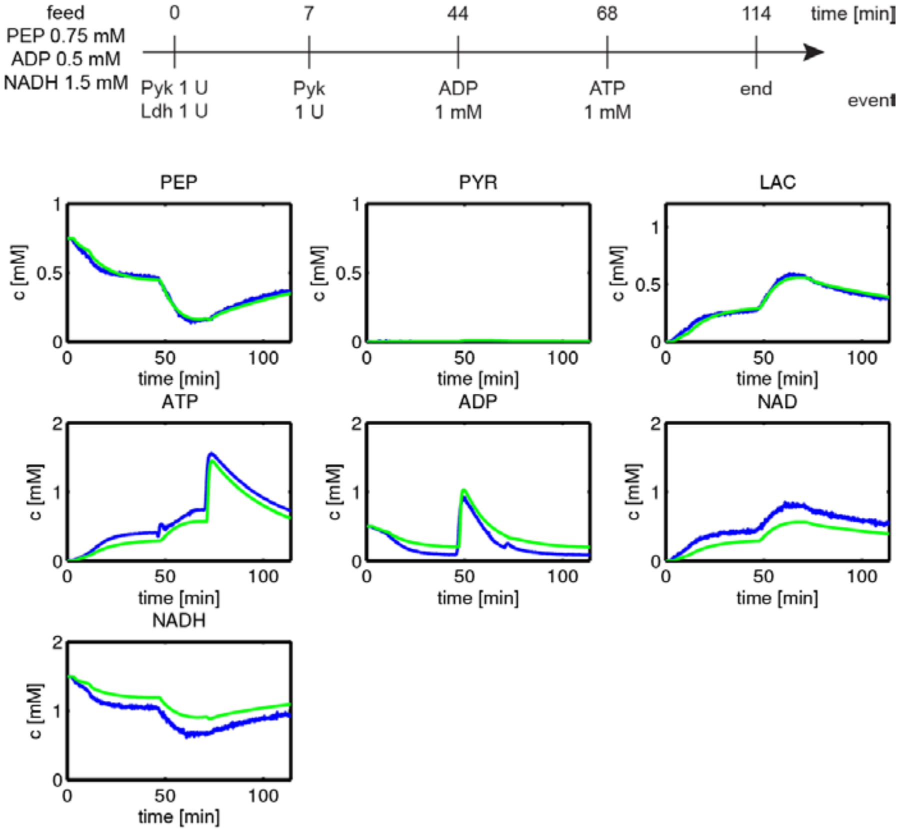
feed
PEP 0.75 mM
ADP 0.5 mM
NADH 1.5 mM
0
7
44
68
114
time [min]
Pyk 1 U Ldh 1 U
Pyk 1 U
ADP 1 mM
ATP 1 mM
end
event
PEP
PYR
LAC
ATP
ADP
NAD
NADH
c [mM]
time [min]

C

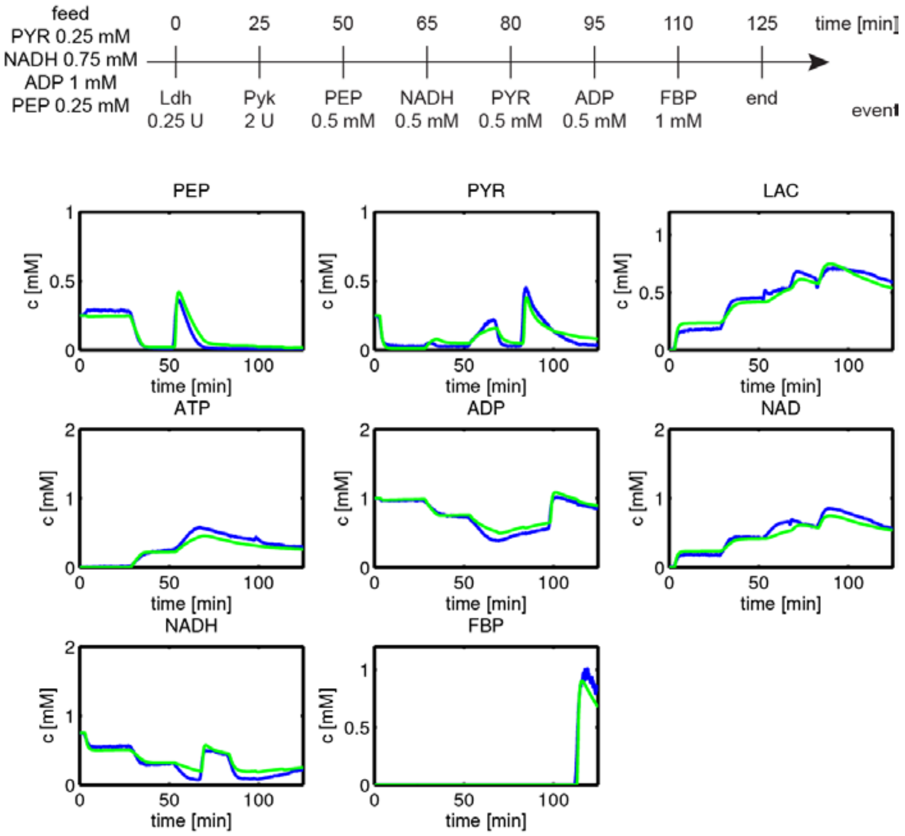
feed
PYR 0.25 mM
NADH 0.75 mM
ADP 1 mM
PEP 0.25 mM
0
25
50
65
80
95
110
125
time [min]
Ldh 0.25 U
Pyk 2 U
PEP 0.5 mM
NADH 0.5 mM
PYR 0.5 mM
ADP 0.5 mM
FBP 1 mM
end
event
PEP
PYR
LAC
ATP
ADP
NAD
NADH
FBP
c [mM]
time [min]

D

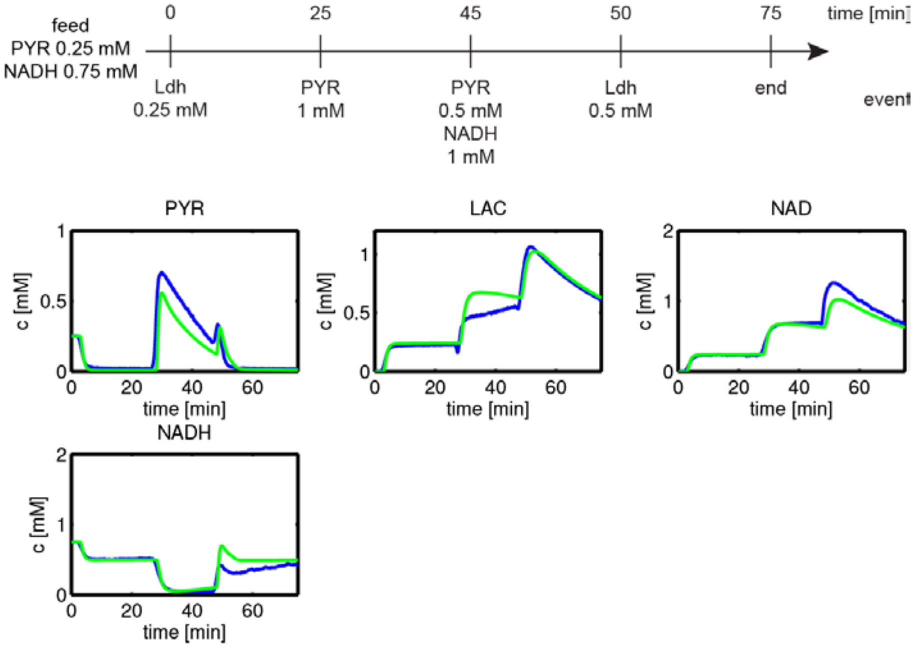
feed
PYR 0.25 mM
NADH 0.75 mM
0
25
45
50
75
time [min]
Ldh 0.25 mM
PYR 1 mM
PYR 0.5 mM NADH 1 mM
Ldh 0.5 mM
end
event
PYR
LAC
NAD
NADH
c [mM]
time [min]

E

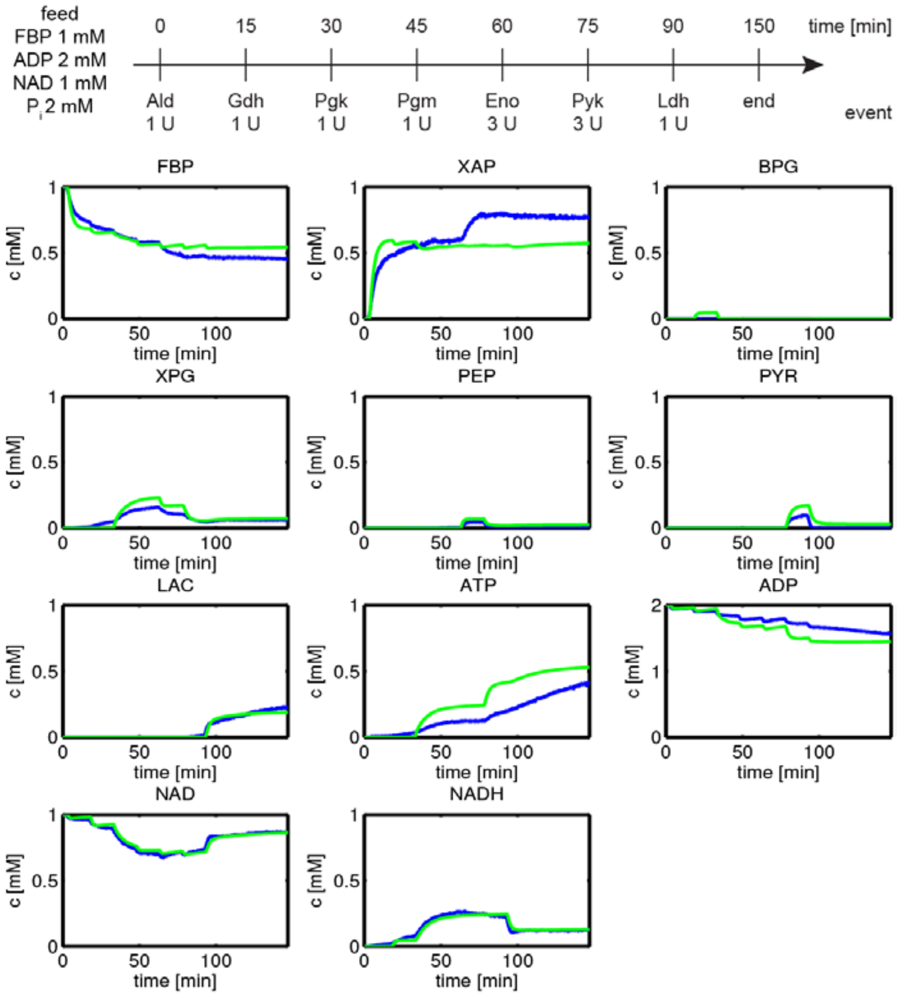

Supplementary Figure 9: Experiments for the parameter estimation of subsystem E, experiment and fit. Each section of the figure represents one of the experiments E1 to E5 and features a summary of the experiment in the upper panel (see also Supplementary Table 12), followed by the concentration time series (measured: blue, best-fit simulation: green). The parameters for the best-fit simulation were obtained from Supplementary Table 13. **A:** E1; **B:** E2; **C:** E3; **D:** E4; **E:** E5. Please note: In experiment E5, the XAP dynamics at 60 min showed an increase in DHAP in response to Eno injection. Until then we never had observed such an effect and also in the experiments in the following sections this type of dynamics never occurred again. Hence we considered this a measurement artefact which we did not follow up.

A

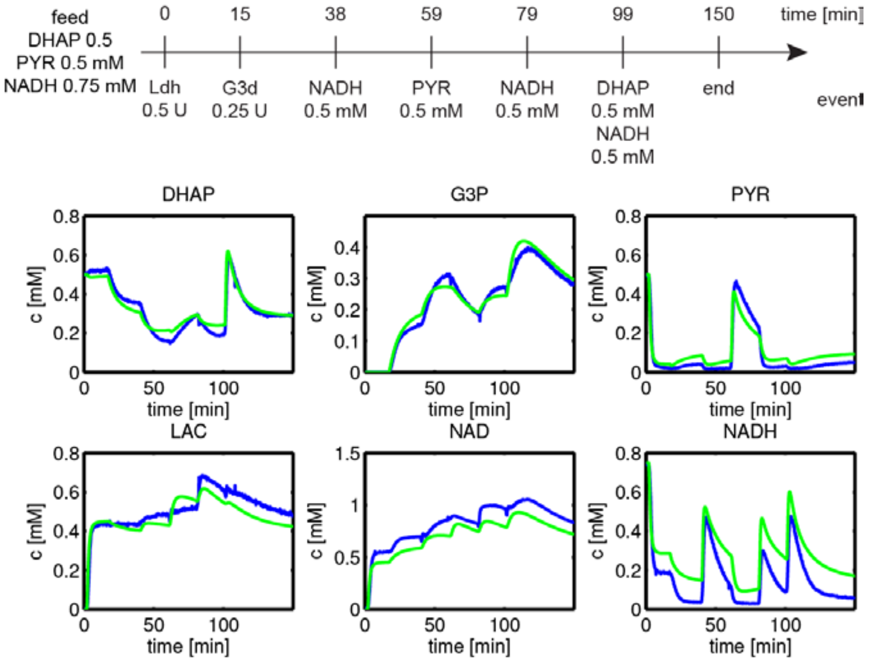

B

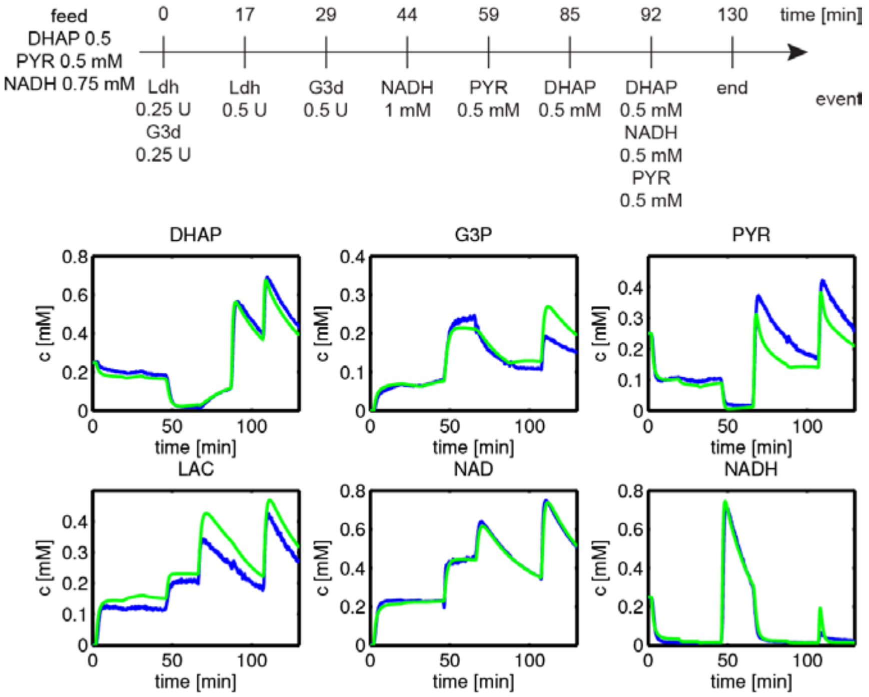

Supplementary Figure 10: Experiments for the parameter estimation of subsystem F, experiment and fit. Each section of the figure represents one of the experiments F1 and F2 E5 and features a summary of the experiment in the upper panel (see also Supplementary Table 14), followed by the concentration time series (measured: blue, best-fit simulation: green). The parameters for the best-fit simulation were obtained from Supplementary Table 15. **A:** F1; **B:** F2.

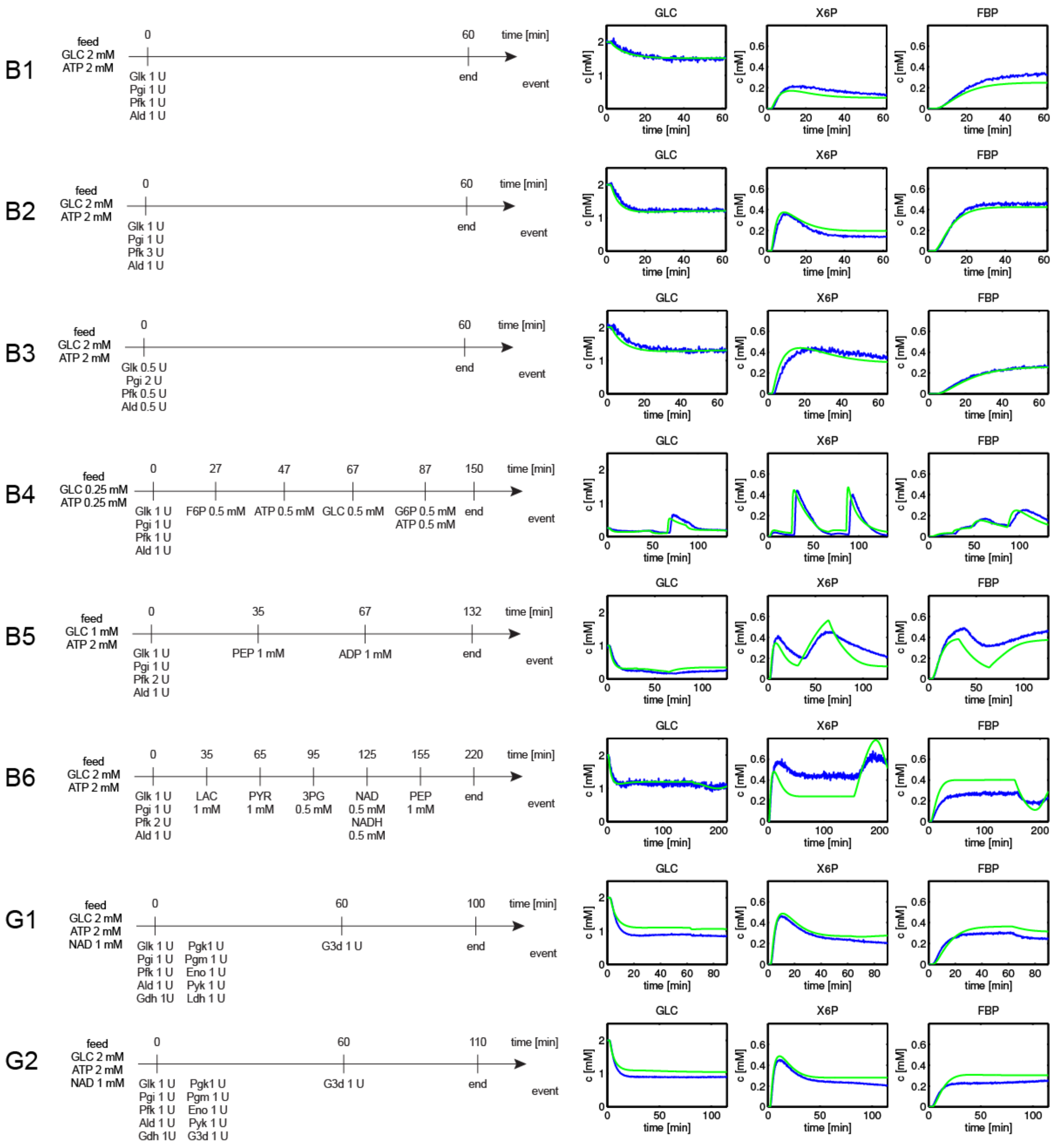

Supplementary Figure 11: Estimation of the parameters of subsystem G, simulation and fit. Estimation of the final set of parameters (Supplementary Table 17) of subsystem G was performed by re-using experiments B1 to B6 from subsystem B and including them in the estimation. Shown are the data for B1 to B6 (experimental data as in Supplementary Table 6) and for experiments G1 and G2 (experimental new data from Supplementary Table 16) and the best-fit simulation with new parameter set from Supplementary Table 17 (for Pfk, Ald) and Supplementary Table 7 (Glk, Pgi)).

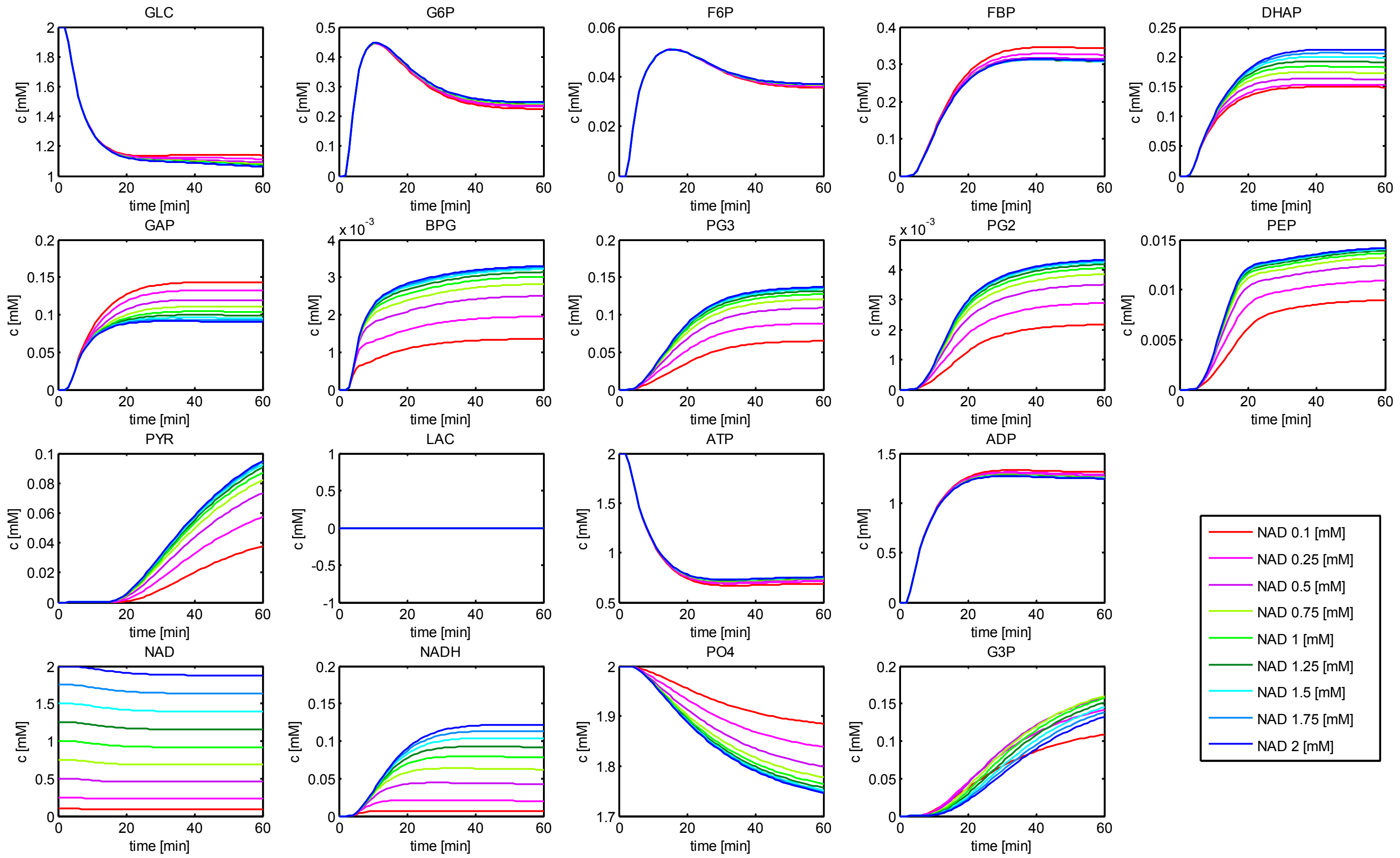

Supplementary Figure 12: Simulated effect of reducing NAD concentration in the feed on performance of complete reaction system in a constant feed experiment on the time courses of compound concentrations. Feed composition: 2 mM GLC, 2 mM ATP and 2 mM P_i plus NAD concentration as indicated in legend. A total of 10 U of enzymes was distributed evenly.

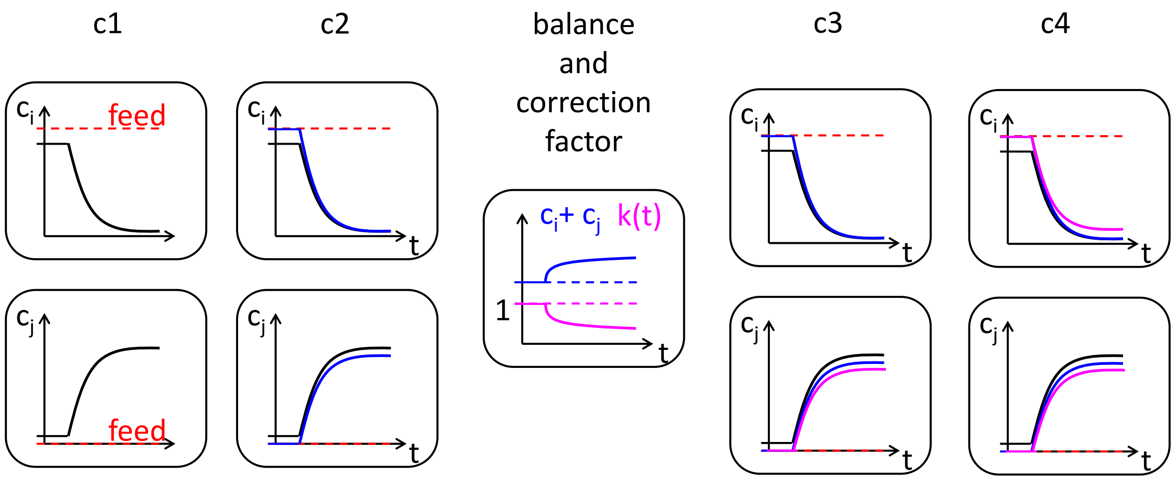

Supplementary Figure 13: Illustration of the data processing after calculation of concentrations from the specific quantification method. Idealized behavior is shown for two compounds, one of which is present in the feed (upper panel), and one of which is not present (equivalent to a feed concentration of 0, lower panel). Data set c1 contains the compound concentrations (black lines) calculated with the help of the different standards after correcting for ADP/ATP crosstalk. Data set c2 contains the compound concentrations after correction of offsets (blue lines). Next, a time dependent correction factor was calculated from the ideal and real mass balances (symbolized by the lines deviating from the constant balance after the enzyme injection in the middle panel). To obtain data set c3, this correction factor was applied to compounds with initial concentrations of zero, resulting in the pink curve (only in the lower panel). In data set c4, the correction factor was additionally applied to the remaining compounds (initial concentration larger than zero), resulting in the pink curve of the upper panel.

Supplementary Tables

Compound abbreviation	Full name	Enzyme abbreviation	Full name
**GLC**	glucose	Glk	glucokinase
**^{13}GLC**	[U-$^{13}C_6$] glucose	Pgi	phosphoglucoisomerase
**G6P**	glucose 6-phosphate	Pfk	phosphofructokinase
**F6P**	fructose 6-phosphate	Ald	fructosebisphosphate aldolase
**X6P**	pool of G6P and F6P	G3d	glycerol 3-phosphate dehydrogenase
**FBP**	fructose 1,6-bisphosphate	Gdh	glyceraldehyde 3-phosphate dehydrogenase
**DHAP**	dihydroxyacetone phosphate	Pgk	phosphoglycerate kinase
**G3P**	glycerol 3-phosphate	Pgm	phosphoglycerate mutase
**GAP**	glyceraldehyde 3-phosphate	Eno	enolase
**XAP**	pool of DHAP and GAP	Pyk	pyruvate kinase
**13BPG**	1,3-bisphosphoglycerate	Ldh	lactate dehydrogenase
**3PG**	3-phosphoglycerate		
**2PG**	2-phosphoglycerate		
**XPG**	pool of 3PG and 2PG		
**PEP**	phosphoenolpyruvate		
**PYR**	pyruvate		
**LAC**	lactate		
**G3P**	glycerol 3-phosphate		
**ATP**	adenosine triphosphate		
**^{15}ATP**	[U-$^{15}N_5$] adenosine triphosphate		
**ADP**	adenosine diphosphate		
**P_i**	inorganic phosphate		
**NAD**	nicotinamide adenine dinucleotide (oxidized form)		
**NADH**	nicotinamide adenine dinucleotide (reduced form)		
**TAU**	taurine		
**^{15}TAU**	[^{15}N] taurine		
**23BPG**	2,3-bisphosphoglycerate		
**HEPES**	4-(2-hydroxyethyl)-1-piperazineethanesulfonic acid		
**MOPS**	4-morpholinepropanesulfonic acid		

Supplementary Table 1: Abbreviations of compounds and enzymes used in the main text. “U” characterizes labeling of all C or N atoms of that compound.

Compound	Q1 m/z	Q3 m/z	DP [V]	CE [V]	CXP [V]
**GLC**	179	89	-25	-12	-13
**X6P**	259	97	-35	-22	-15
**FBP**	339	97	-30	-30	-15
**XAP**	169	97	-25	-14	-15
**BPG**	265	167	-40	-20	-13
**XPG**	185	97	-25	-20	-15
**PEP**	167	79.1	-20	-14	-11
**PYR**	87	43.1	-20	-12	-5
**LAC**	89	43.2	-25	-18	-5
**G3P**	171	79.1	-35	-22	-11
**ATP**	506	408	-50	-32	-19
**ADP**	426	158.9	-50	-38	-25
**NAD**	662	540.1	-30	-18	-29
**NADH**	664	346	-90	-48	-17
**^{13}GLC**	185	92	-25	-12	-13
**^{13}X6P**	265	97	-35	-22	-15
**^{13}FBP**	345	97	-30	-30	-15
**^{13}XAP**	172	97	-25	-14	-15
**^{13}BPG**	268	170	-40	-20	-13
**^{13}XPG**	188	97	-25	-20	-15
**^{13}PEP**	170	79.1	-20	-14	-11
**^{13}PYR**	90	45.1	-20	-12	-5
**^{13}LAC**	92	45.2	-25	-18	-5
**^{13}G3P**	174	79.1	-35	-22	-11
**^{15}ATP**	511	413	-50	-32	-19
**^{15}ADP**	431	158.9	-50	-38	-25
**TAU**	124.2	79.9	-55	-26	-11
**^{15}TAU**	125.2	79.9	-55	-26	-11

Supplementary Table 2: Mass spectrometry settings for all measured compounds. Mass to charge ratio of the principal ion, the selected fragment and optimized instrument parameters. DP: declustering potential; CE: collision energy; CXP: collision cell exit potential.

Enyzme	Original host	Source	Product number	Unit definition		
				pH	Tmp. [°C]	Direction
**Glk**	*E. coli*	custom produced, see Materials and Methods section	-	7.8	30	Forward
**Pgi**	Yeast	Sigma-Aldrich	P5381	7.4	25	reverse
**Pfk**	*Bacillus stearothermophilus*	Sigma-Aldrich	F0137	9.0	30	forward
**Ald**	rabbit muscle	Sigma-Aldrich	A8811	7.4	25	forward
**G3d**	rabbit muscle	Roche	10127752001	N/A	25	forward
**Gdh**	yeast	Sigma-Aldrich	G5537	7.6	25	reverse
**Pgk**	yeast	Sigma-Aldrich	P7634	6.9	25	forward
**Pgm**	recombinant human	Creative Biomart	PGAM1-2485TH	7.8	30	forward
**Eno**	yeast	Sigma-Aldrich	E6126	7.4	25	forward
**Pyk**	rabbit muscle	Sigma-Aldrich	P1506	7.6	37	forward
**Ldh**	*E. coli*	Sigma-Aldrich	59747	7.4	25	forward

Supplementary Table 3: Commercial sources of used enzymes and key elements of the definition of activity applied by the supplier. One unit of activity corresponds to the formation of 1 µmol min^{-1} of product at the given pH and temperature for the reaction for which the enzyme is responsible in the glycolysis. According to the product description the rate was either determined in the direction of standard glycolysis (“forward”) or in the opposite direction (“reverse”).

System	Type of experiment	#	Concentration of compounds in feed 1 [mM]	Concentration of compounds in feed 2 [mM]	Initial enzyme activity [U]	Time [min]	Event
**A**		A1	GLC 1.2<br>ATP 1.4<br>$MgCl_2$ 2.5	-	Glk 0.42	30	end
	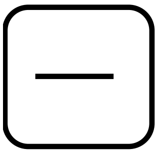	A2	GLC 2.6<br>ATP 8.4<br>$MgCl_2$ 10.5	-	Glk 0.42	30	end
		A3	GLC 1.3<br>ATP 4.3<br>$MgCl_2$ 6.3	-	Glk 0.42	30	end
		A4	GLC 1.7<br>ATP 0.5<br>$MgCl_2$ 4.9	GLC 1.7<br>ATP 2.9<br>$MgCl_2$ 4.9	Glk 0.42	<0<br>0<br>60	100% feed 1<br>100% feed 2<br>end
	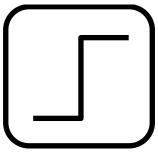	A5	GLC 7.2<br>ATP 0.4<br>$MgCl_2$ 4.9	GLC 0<br>ATP 2.7<br>$MgCl_2$ 4.9	Glk 0.42	<0<br>0<br>60	100% feed 1<br>100% feed 2<br>end
		A6	GLC 0.1<br>ATP 2.8<br>$MgCl_2$ 3.8	GLC 0.9<br>ATP 0.8<br>$MgCl_2$ 3.8	Glk 0.42	<0<br>0<br>60	100% feed 1<br>100% feed 2<br>end
		A7	GLC 0.4<br>ATP 7.5<br>$MgCl_2$ 9.0	GLC 8.0<br>ATP 3.3<br>$MgCl_2$ 9.0	Glk 0.42	0<br>60<br>120	100% feed 1<br>100% feed 2<br>end
	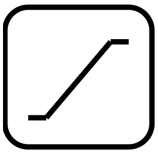	A8	GLC 1.0<br>ATP 0.5<br>$MgCl_2$ 4.0	GLC 1.0<br>ATP 3.0<br>$MgCl_2$ 4.0	Glk 0.42	0<br>60<br>120	100% feed 1<br>100% feed 2<br>end
		A9	GLC 0.8<br>ATP 1.7<br>$MgCl_2$ 4.0	GLC 1.7<br>ATP 3.1<br>$MgCl_2$ 4.0	Glk 0.42	0<br>60<br>120	100% feed 1<br>100% feed 2<br>end

Supplementary Table 4: Experiments for the parameterization of subsystem A. Indicated are the type of experiments (first column, “Type of experiment”); an i nternal identifier of the specific experiment (second column); the composition of feed 1 (provided from the first HPLC pump; please note that the composition of feed 1 is equal to the initial reactor concentration, third column); the composition of feed 2 (required to produce gradients, all of which were linear, fourth column); the amount of enzyme injected at t=0 to start the experiment (fifth column); time points of events (sixth column); and the nature of the events (seventh column). $MgCl_2$ was provided in all cases in excess of the maximal ATP concentration in the feed.

Enzyme	Parameter	Unit	Lower boundary	Best estimate	Upper boundary
**Glk**	V_{max}	mM min^{-1}	0.0715	0.204	0.286
	K_G	mM	0.0001	0.704	4
	K_{ATP}	mM	0.0001	0.493	4
	$K_{i,ADP}$	mM	0.0001	0.306	4

Supplementary Table 5: Estimated parameter values of subsystem A. Parameters are shown with their corresponding lower and upper boundaries used during optimization.

System	Type of experiment	#	Concentration of compounds in feed [mM]	Initial enzyme activity [U]	Time [min]	Pulse [mM] (equivalent to reactor concen-
B		B1	GLC 2<br>ATP 2	Glk 1<br>Pgi 1<br>Pfk 1<br>Ald 1	60	end
	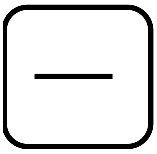	B2	GLC 2<br>ATP 2	Glk 1<br>Pgi 1<br>Pfk 3<br>Ald 1	60	end
		B3	GLC 2<br>ATP 2	Glk 0.5<br>Pgi 2<br>Pfk 0.5<br>Ald 0.5	60	end
		B4	GLC 0.25<br>ATP 0.25	Glk 1<br>Pgi 1<br>Pfk 1<br>Ald 1	27<br>47<br>67<br>87<br><br>150	F6P 0.5<br>ATP 0.5<br>GLC 0.5<br>G6P 0.5<br>ATP 0.5<br>end
	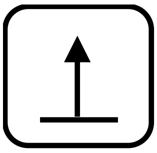	B5	GLC 1<br>ATP 2<br>$MgCl_2$ 4	Glk 1<br>Pgi 1<br>Pfk 2<br>Ald 1	35<br>67<br>132	PEP 1<br>ADP 1<br>end
		B6	GLC 2<br>ATP 2	Glk 1<br>Pgi 1<br>Pfk 2<br>Ald 1	35<br>65<br>95<br>125<br><br>155<br>220	LAC 1<br>PYR 1<br>3PG 0.5<br>NAD 0.5<br>NADH 0.5<br>PEP 1<br>end

Supplementary Table 6: Experiments for the parameterization of subsystem B. Indicated are experimental type, number, feed concentrations of compounds (equals initial concentration in the reactor), injected amount of enzyme at t=0 and timing of pulses. For compound pulses the (theoretical) reactor concentration and for enzyme pulses the absolute activity added to the reactor are given. If not otherwise stated the $MgCl_2$ was 2.5 mM.

Enzyme	Parameter	Unit	Lower boundary	Best estimate	Upper boundary
**Glk**	V_{max}	mM min^{-1}	0.0715	0.286	0.286
	K_G	mM	0.0001	0.413	4
	K_{ATP}	mM	0.0001	0.454	4
	$K_{i,ADP}$	mM	0.0001	0.160	4
**Pgi**	V_{max}	mM min^{-1}	0.0715	0.108	0.286
	K^{eq}	-	-	0.28	-
	K_{G6P}	mM	-	0.2	-
	K_{F6P}	mM	-	0.2	-
**Pfk**	V_{max}	mM min^{-1}	0.0715	0.0908	0.286
	K_{ATP}	mM	0.0001	0.0216	4
	$K_{i,ADP}$	mM	0.0001	0.00880	4
	K_{F6P}	mM	0.0001	0.000561	4
	$K_{T,PEP}$	mM	0.0001	0.0797	4
	$K_{R,ADP}$	mM	0.0001	2.52	4
	L_0	-	4.00E+04	4.00E+06	4.00E+08
	c	-	0.000005	0.00210	0.05
	n	-	3	4.00	4
**Ald**	V_{max}	mM min^{-1}	0.0715	0.131	0.286
	K^{eq}	mM	-	0.036	-
	K_{FBP}	mM	-	0.12	-
	K_{DHAP}	mM	-	0.088	-
	K_{GAP}	mM	-	0.088	-

Supplementary Table 7: Estimated parameter values of subsystem B. Parameters are shown with their corresponding lower and upper boundaries used during optimization. Parameters without specified boundaries were taken from literature or calculated separately and were not estimated in these experiments (see text for details).

Sys-tem	Type of experiment	#	Concen-tration of compounds in feed [mM]	Initial enzyme activity [U]	Time [min]	Pulse [mM] (equivalent to reactor concen-tration), [U]
**C**	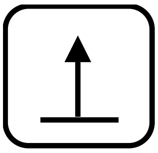	C1	FBP 0.5<br>ADP 0.5<br>NAD 0.5<br>P_i 0.5	Ald 1<br>Gdh 0.5<br>Pgk 0.5	40<br>51<br>63<br>74<br>92<br>133	NAD 0.5<br>ADP 0.5<br>P_i 0.5<br>FBP 0.5<br>G3d 0.5<br>end
	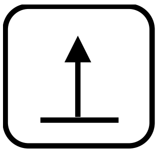	C2	FBP 1<br>ADP 1.5<br>NAD 1<br>P_i 2	Ald 0.5<br>Gdh 1<br>Pgk 1	30<br>45<br>60<br>75<br>90<br>105<br>120<br>135<br>155	Gdh 1<br>Ald 0.5<br>Pgk 1<br>NAD 1<br>ADP 1<br>FBP 1<br>NADH 1<br>P_i 1<br>end
	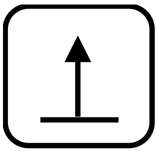	C3 (data from E5)	FBP 1<br>ADP 2<br>NAD 1<br>P_i 2	Ald 1	15<br>30<br>45	Gdh 1<br>Pgk 1<br>end
	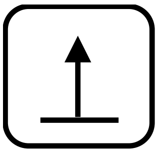	C4	DHAP 1<br>NADH 0.5	G3d 0.25	20<br>40<br>60<br><br>80<br>120	NADH 1<br>DHAP 1<br>DHAP 0.5<br>NADH 1<br>G3d 0.25<br>end

Supplementary Table 8: Experiments for the parameterization of subsystem C. Indicated are experimental type, number, feed concentrations of compounds (equals initial concentration in the reactor), injected amount of enzyme at t=0 and timing of pulses. For compound pulses the (theoretical) reactor concentration and for enzyme pulses the absolute activity added to the reactor are given.

Enzyme	Parameter	Unit	Lower boundary	Best estimate	Upper boundary
**Ald**	V_{max}	mM min^{-1}	0.143	0.143	0.572
	K^{eq}	mM	0.00005	0.170	1
	K_{FBP}	mM	0.00042	0.320	0.32
	K_{DHAP}	mM	0.2	0.267	4
	K_{GAP}	mM	0.15	0.150	2
**Gdh**	V_{max}	mM min^{-1}	0.143	0.204	0.572
	K^{eq}	mM^{-1}	0.0032	0.00501	9.3
	K_{NAD}	mM	0.0001	0.00889	2
	K_{GAP}	mM	0.0001	1.43	2
	K_{Pi}	mM	0.0001	0.000305	2
	K_{NADH}	mM	0.0001	0.00928	2
	K_{BPG}	mM	0.0001	0.0659	2
	n	-	2	2.00	4
**Pgk**	V_{max}	mM min^{-1}	0.143	0.143	0.572
	K^{eq}	-	11.5	50.9	56
	K_{BPG}	mM	5.00E-07	0.0161	0.2
	K_{ADP}	mM	0.05	0.0500	1
	K_{PG3}	mM	0.385	2.56	2.56
	K_{ATP}	mM	0.165	0.165	0.84
**G3d**	V_{max}	mM min^{-1}	0.143	0.183	0.572
	K_{DHAP}	mM	0.0001	1.43	2
	K_{NADH}	mM	0.0001	0.0001	2
	K_{NAD}	mM	0.0001	1.29	2

Supplementary Table 9: Estimated parameter values of subsystem C. Parameters are shown with their corresponding lower and upper boundaries used during optimization. Parameters without specified boundaries were taken from literature or calculated separately and were not estimated in these experiments (see text for details).

System	Type of experiment	#	Concentration of compounds in feed [mM]	Initial enzyme activity [U]	Time [min]	Pulse [mM] (equivalent to reactor concentration), [U]
**D**	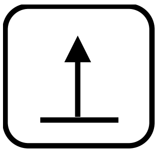	D1	3PG 0.5	Pgm 0.875<br>Eno 0.5	15<br>35<br>50<br>70<br>109<br>131<br>159<br>171<br>177	3PG 1<br>Pgm 0.875<br>PYR 1<br>Eno 0.5<br>Eno 5<br>Pgm 1.25<br>PEP 1<br>Eno 0.5<br>end
	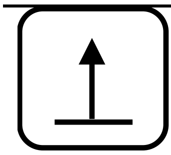	D2	3PG 0.5	Pgm 0.4375<br>Eno 1	30<br>61<br>92<br>146	PEP 0.5<br>Pgm 0.4375<br>3PG 0.5<br>end
	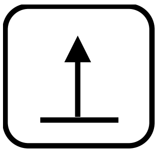	D3 (data from E5)	FBP 1<br>ADP 2<br>NAD 1<br>P_i 2	Ald 1	15<br>30<br>45<br>60	Gdh 1<br>Pgk 1<br>Pgm 1<br>end

Supplementary Table 10: Experiments for the parameterization of subsystem D. Indicated are experimental type, number, feed concentrations of compounds (equals initial concentration in the reactor), injected amount of enzyme at t=0 and timing of pulses. For compound pulses the (theoretical) reactor concentration and for enzyme pulses the absolute activity added to the reactor are given.

Enzyme	Parameter	Unit	Lower boundary	Best estimate	Upper boundary
Pgm	V_{max}	mM min^{-1}	0.143	0.143	0.572
	K^{eq}	mM	0.0435	0.0435	0.2
	K_{PG3}	mM	0.11	0.800	0.8
	K_{PG2}	mM	0.05	0.0500	1
Eno	V_{max}	mM min^{-1}	0.143	0.513	0.572
	K^{eq}	-	1.8	5.64	10.2
	K_{PG2}	mM	0.0215	0.0458	0.6
	K_{PEP}	mM	0.005	0.00500	1

Supplementary Table 11: Estimated parameter values of system D. Parameters are shown with their corresponding lower and upper boundaries used during optimization. Parameters without specified boundaries were taken from literature or calculated separately and were not estimated in these experiments (see text for details).

Sys-tem	Type of experiment	#	Concen-tration of compounds in feed [mM]	Initial enzyme activity [U]	Time [min]	Pulse [mM] (equivalent to reactor concen-tration), [U]
**E**	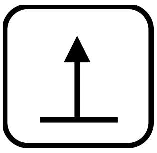	E1	PEP 0.5	Pyk 4.35	22	PEP 1
			ADP 1	Ldh 1	37	ADP 0.5
			NADH 1		52	NADH 0.5
					67	PYR 0.5
					82	FBP 0.5
					92	NADH 0.5
						PEP 0.45
						ADP 0.5
					132	end
	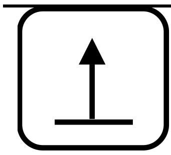	E2	PEP 0.75	Pyk 1	7	Pyk 1
			ADP 0.5	Ldh 1	44	ADP 1
			NADH 1.5		68	ATP 1
					114	end
	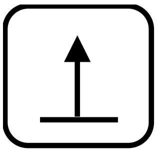	E3	PYR 0.25	Ldh 0.25	25	Pyk 2
			ADP 1		50	PEP 0.5
			NADH 0.75		65	NADH 0.5
			PEP 0.25		80	PYR 0.5
					95	ADP 0.5
					110	FBP 1
					125	end
	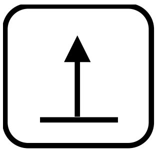	E4	PYR 0.25	Ldh 0.5	25	PYR 1
			NADH 0.75		45	PYR 0.5
						NADH 1
					50	Ldh 0.5
					75	end
	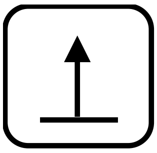	E5	FBP 1	Ald 1	15	Gdh 1
			ADP 2		30	Pgk 1
			NAD 1		45	Pgm 1
			P_i 2		60	Eno 3
					75	Pyk 3
					90	Ldh 1
					150	end

Supplementary Table 12: Experiments for the parameterization of subsystem E. Indicated are experimental type, number, feed concentrations of compounds (equals initial concentration in the reactor), injected amount of enzyme at t=0 and timing of pulses. For compound pulses the (theoretical) reactor concentration and for enzyme pulses the absolute activity added to the reactor are given.

Enzyme	Parameter	Unit	Lower boundary	Best estimate	Upper boundary
Pyk	V_{max}	mM min^{-1}	0.03575	0.0621	0.572
	$K_{R,PEP}$	mM	0.0001	0.000223	2
	K_{ADP}	mM	0.0001	1.99	2
	$K_{R,ATP}$	mM	0.0001	0.101	2
	$K_{T,FBP}$	mM	0.0001	2.00	2
	L_0	-	1.00E+04	2.19E+05	1.00E+06
	n	-	3	3.24	4
Ldh	V_{max}	mM min^{-1}	0.143	0.396	0.572
	K^{eq}	-	270	507	2000
	K_{PYR}	mM	0.016	0.0168	0.2
	K_{NADH}	mM	0.007	0.2000	0.2
	K_{NAD}	mM	0.015	0.0150	0.1

Supplementary Table 13: Estimated parameter values of system E. Parameters were obtained from experiments E1 to E5. Parameters are shown with their corresponding lower and upper boundaries used during optimization. Parameters without specified boundaries were taken from literature or calculated separately and w ere not estimated in these experiments (see text for details).

System	Type of experiment	#	Concentration of compounds in feed [mM]	Initial enzyme activity [U]	Time [min]	Pulse [mM] (equivalent to reactor concentration), [U]
**F**	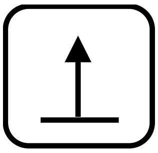	F1	DHAP 0.5<br>PYR 0.5<br>NADH 0.75	Ldh 0.5	15<br>38<br>59<br>79<br>99<br>150	G3d 0.25<br>NADH 0.5<br>PYR 0.5<br>NADH 0.5<br>DHAP 0.5, NADH 0.5<br>end
	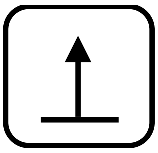	F2	DHAP 0.25<br>PYR 0.25<br>NADH 0.25	Ldh 0.25<br>G3d 0.25	17<br>29<br>44<br>59<br>85<br>105<br><br>130	Ldh 0.5<br>G3d 0.5<br>NADH 1<br>PYR 0.5<br>DHAP 0.5<br>DHAP 0.5, NADH 0.5, PYR 0.5<br>end

Supplementary Table 14: Experiments for the parameterization of subsystem F. Indicated are experimental type, number, feed concentrations of compounds (equals initial concentration in the reactor), injected amount of enzyme at t=0 and timing of pulses. For compound pulses the (theoretical) reactor concentration and for enzyme pulses the absolute activity added to the reactor are given.

Enzyme	Parameter	Unit	Lower boundary	Best estimate	Upper boundary
Pyk	V_{max}	mM min^{-1}	0.03575	0.157	0.572
	$K_{R,PEP}$	mM	0.0001	0.000100	2
	K_{ADP}	mM	0.0001	6.00	6
	$K_{R,ATP}$	mM	0.0001	0.0674	2
	$K_{T,FBP}$	mM	0.0001	0.230	6
Ldh	V_{max}	mM min^{-1}	0.03575	0.569	0.572
	K_{PYR}	mM	0.0001	0.0196	2
	K_{NADH}	mM	0.0001	0.0200	0.2
	K_{NAD}	mM	0.0001	0.00933	2
G3d	V_{max}	mM min^{-1}	0.03575	0.150	0.572
	K_{DHAP}	mM	0.0001	0.0527	2
	K_{NADH}	mM	0.0001	0.00899	0.02
	K_{NAD}	mM	0.0001	0.00143	2

Supplementary Table 15: Estimated parameter values of system F. Parameters were obtained from experiments E1 to F2**.** Parameters are shown with their corresponding lower and upper boundaries used during optimization. Parameters without specified boundaries were taken from literature or calculated separately and w ere not estimated in these experiments (see text for details).

Sys-tem	Type of experiment	#	Concen-tration of compounds in feed [mM]	Initial enzyme activity [U]	Time [min]	Pulse [mM] (equivalent to reactor concen-tration), [U]
**G**		B1-6	see Supplementary Table 6			
	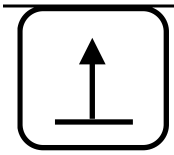	G1	GLC 2<br>ATP 2<br>NAD 1<br>P_i 2	Glk 1<br>Pgi 1<br>Pfk 1<br>Ald 1<br>Gdh 1<br>Pgk 1<br>Pgm 1<br>Eno 1<br>Pyk 1<br>Ldh 1	60<br>100	G3d 1<br>end
	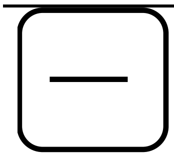	G2	GLC 2<br>ATP 2<br>NAD 1<br>P_i 2	Glk 1<br>Pgi 1<br>Pfk 1<br>Ald 1<br>Gdh 1<br>Pgk 1<br>Pgm 1<br>Eno 1<br>Pyk 1<br>G3d 1	110	end

Supplementary Table 16: Experiments for the parameterization of subsystem G. Indicated are experimental type, number, feed concentrations of compounds (equals initial concentration in the reactor), injected amount of enzyme at t=0 and timing of pulses. For compound pulses the (theoretical) reactor concentration and for enzyme pulses the absolute activity added to the reactor are given.

Enzyme	Parameter	Unit	Lower boundary	Best estimate	Upper boundary
**Pfk**	V_{max}	mM min^{-1}	0.143	0.528	0.572
	K_{ATP}	mM	0.0001	0.000457	2
	$K_{i.ADP}$	mM	0.0001	0.000774	2
	K_{F6P}	mM	0.0001	0.3704	2
	$K_{T,PEP}$	mM	0.0001	0.00330	2
	$K_{R,ADP}$	mM	0.0001	0.000765	2
**Ald**	V_{max}	mM min^{-1}	0.143	0.570	0.572
	K^{eq}	mM	5.00E-05	0.0630	2
	K_{FBP}	mM	0.0001	0.000318	2
	K_{DHAP}	mM	0.0001	1.61	2
	K_{GAP}	mM	0.0001	1.02	2

Supplementary Table 17: Estimated parameter values of subsystem G. Parameters were obtained from experiments B1 to B6 and G1 and G2 with their corresponding lower and upper bounds. Parameters without specified boundaries were taken from literature or calculated separately and were not estimated in these experiments (see text for details).

Enzyme	Host E.C. number	Parameter	Unit	Lower boundary	Best estimate	Upper boundary	Host range	General range	Source Supplementary Table
**Glk**	E. coli 2.7.1.2	V_{max}	mM min^{-1}	0.0715	0.286	0.286			7
		K_G	mM	0.0001	0.413	4	0.15 - 0.78	0.028 - 7.7	7
		K_{ATP}	mM	0.0001	0.454	4	0.5 - 3.76	0.05 - 3.76	7
		$K_{i,ADP}$	mM	0.0001	0.160	4	-	-	7
**Pgi**	yeast 5.3.1.9	V_{max}	mM min^{-1}	0.0715	0.108	0.286			7
		K^{eq}	-	-	0.28	-			7
		K_{G6P}	mM	-	0.2	-	0.3 - 1.5	0.11-11.7	7
		K_{F6P}	mM	-	0.2	-	0.11 - 0.23	0.031 - 1	7
**Pfk**	B. stearo-thermophilus 2.7.1.11	V_{max}	mM min^{-1}	0.143	0.528	0.572			17
		K_{ATP}	mM	0.0001	0.000457	2	-	0.005 - 0.7	17
		$K_{i,ADP}$	mM	0.0001	0.000774	2	-	1.75 - 2.1	17
		K_{F6P}	mM	0.0001	0.3704	2	-	0.007 - 7	17
		$K_{T,PEP}$	mM	0.0001	0.00330	2	-	0.0272 - 0.83	17
		$K_{R,ADP}$	mM	0.0001	0.000765	2	-	-	17
		L_0	-	4.00e4	4.00e6	4.00e8			7
		c	-	0.000005	0.00210	0.05			7
		n	-	3	4.00	4			7
**Ald**	rabbit 4.1.2.13	V_{max}	mM min^{-1}	0.143	0.570	0.572			17
		K^{eq}	mM	5.00e-5	0.0630	2			17
		K_{FBP}	mM	0.0001	0.000318	2	8.4e-4 - 2	1.6e-4 - 4.2	17
		K_{DHAP}	mM	0.0001	1.61	2	0.4 - 2	0.065 - 2	17
		K_{GAP}	mM	0.0001	1.02	2	0.3 - 1	0.057 - 1	17
**Gdh**	yeast 1.2.1.12	V_{max}	mM min^{-1}	0.143	0.204	0.572			9
		K^{eq}	mM^{-1}	0.0032	0.00501	9.3			9
		K_{NAD}	mM	0.0001	0.00889	2	0.1	3.2e-5 - 0.45	9

		K_{GAP}	mM	0.0001	1.43	2	0.6	0.0207 - 1.78	9
		K_{Pi}	mM	0.0001	0.000305	2	1.5	0.2 - 37	9
		K_{NADH}	mM	0.0001	0.00928	2	-	0.0033 - 0.061	9
		K_{BPG}	mM	0.0001	0.0659	2	-	0.002 - 0.47	9
		n	-	2	2.00	4			9
**Pgk**	yeast 2.7.2.3	V_{max}	mM min^{-1}	0.143	0.143	0.572			9
		K^{eq}	-	11.5	50.9	56			9
		K_{BPG}	mM	5.00e-7	0.0161	0.2	-	5e-4 - 0.29	9
		K_{ADP}	mM	0.05	0.0500	1	0.2 - 0.5	0.039 - 7.4	9
		K_{PG3}	mM	0.385	2.56	2.56	0.77 - 1.28	0.0022 - 7.6	9
		K_{ATP}	mM	0.165	0.165	0.84	0.04 - 0.42	0.017 - 9.7	9
**G3d**	rabbit 1.1.1.8	V_{max}	mM min^{-1}	0.0357	0.150	0.572			15
		K_{DHAP}	mM	0.0001	0.0527	0.4	0.075 - 0.46	0.012 - 0.54	15
		K_{NADH}	mM	0.0001	0.00899	0.02	0.0043 - 0.022	0.001 - 0.0905	15
		K_{NAD}	mM	0.0001	0.00143	2	0.0044 - 0.38	0.0044 - 0.65	15
**Pgm**	human 5.4.2.1	V_{max}	mM min^{-1}	0.143	0.143	0.572			11
		K^{eq}	mM	0.0435	0.0435	0.2			11
		K_{PG3}	mM	0.11	0.800	0.8	0.22 - 0.4	0.22 - 6	11
		K_{PG2}	mM	0.05	0.0500	1	-	0.041 - 0.69	11
**Eno**	yeast 4.2.1.11	V_{max}	mM min^{-1}	0.143	0.513	0.572			11
		K^{eq}	-	1.8	5.64	10.2			11
		K_{PG2}	mM	0.0215	0.0458	0.6	0.043 - 0.68	0.025 - 0.71	11
		K_{PEP}	mM	0.005	0.00500	1	-	0.07 - 0.95	11
**Pyk**	rabbit 2.7.1.40	V_{max}	mM min^{-1}	0.0357	0.157	0.572			15
		$K_{R,PEP}$	mM	0.0001	0.000100	2	-	0.0003 - 2.1	15
		K_{ADP}	mM	0.0001	6.00	6	-	0.03 - 2.3	15
		$K_{R,ATP}$	mM	0.0001	0.0674	2	-	-	15
		$K_{T,FBP}$	mM	0.0001	0.230	6	-	-	15

		L_0	-	1.00e4	2.19e5	1.00e6			13
		n	-	3	3.24	4			13
**Ldh**	E. coli 1.1.1.27	V_{max}	mM min^{-1}	0.0357	0.569	0.572			15
		K^{eq}	-	-	507	-			13
		K_{PYR}	mM	0.016	0.0196	1	-	0.016 - 0.5	15
		K_{NADH}	mM	0.007	0.200	0.2	-	0.007 - 0.5	15
		K_{NAD}	mM	0.015	0.00933	2	-	0.0081 - 1	15

Supplementary Table 18: Final set of estimated parameters. Values of the identified parameters with used parameter boundaries and compared to ranges for K_M values obtained from BRENDA [19] for either the specific host ("Host range") or a set of hosts ("General range"). Parameters without indicated boarders were not estimated. Not considered in the range analysis were parameters measured for mutated or immobilized enzymes as well as singular extreme values. Green indicates K_M values within host range and bl ue K_M values inside the general range. Pink indicates that the estimated K_M-value was identical with either the upper or the lower set boundary. Orange indicates that the estimated K_M-value was out of range of the general range of K_M-values obtained from BRENDA.

Enzyme	Starting point [U]<br>-	Optimized [U]<br>Experiment O1
Glk	1	**0.82**
Pgi	1	**0.74**
Pfk	1	**1.44**
**Total**	**3**	**3**

Supplementary Table 19: Optimized distribution of 3 U of enzyme activities for upper glycolysis. The design included an experimental time of 60 min, feed concentrations of 2 mM GLC, 2 mM ATP and initial pulses of the required enzymes to the reactor.

Enzyme	Starting point [U] Experiment S2	Optimization [U] Experiment O2
**Ald**	1	2
**Gdh**	1	2
**Pgk**	1	1.1
**Pgm**	1	0.1
**Eno**	1	0.1
**Pyk**	1	0.1
**G3d**	1	2.6
**Total**	**7**	**7**

Supplementary Table 20: Optimized distribution of 7 U of enzyme activities for lower glycolysis with G3d. The design included an ex perimental time of 80 min, feed concentrations of 1 mM FBP, 2 mM ADP, 1 mM NAD and 2 mM P_i, and initial pulses of the required enzymes to the reactor.

Enzyme	Starting point [U] Experiment S3	Optimized [U] Experiment O3	Starting point [U] -	Optimized [U] Experiment O4
**Glk**	2	3.3	4	6.2
**Pgi**	2	1.8	4	2.7
**Pfk**	2	2.4	4	4.1
**Ald**	2	0.5	4	0.9
**Gdh**	2	3.8	4	6.6
**Pgk**	2	0.7	4	4.0
**Pgm**	2	0.5	4	0.9
**Eno**	2	1.5	4	2.7
**Pyk**	2	2.2	4	3.6
**G3d**	2	3.2	4	8.3
**Total**	**20**	**20**	**40**	**40**

Supplementary Table 21: Optimized full reaction system. Activity distributions of the G3P production system as used for equi-activity reference and optimized experiment of 20 U and optimized 40 U experiment. The design included an experimental time of 60 min, feed concentrations of 2 mM GLC, 2 mM ATP, 1 mM NAD and 2 mM P_i, and initial pulses of the required enzymes to the reactor.

Enzyme	Starting point [U]	Optimization[U] Experiment O5
**Glk**	1	1.7
**Pgi**	1	1
**Pfk**	1	1.2
**Ald**	1	0.3
**Gdh**	1	1.3
**Pgk**	1	1.1
**Pgm**	1	0.5
**Eno**	1	0.5
**Pyk**	1	0.5
**G3d**	1	2
**Total**	**10**	**10**

Supplementary Table 22: Experimental conditions for optimization of NAD requirement. Catalytically relevant compound concentrations in the reactor feed and enzymatic activity injected into the reactor are given.

Enzyme	EC number	Reaction in glycolytic direction	Reaction equilibrium for different ionic strengths	
			0M	0.1M
**Glk**	2.7.1.2	GLC + ATP = G6P + ADP	1.1e5	8.6e4
**Pgi**	5.3.1.9	G6P = F6P	2.8e-1	2.8e-1
**Pfk**	2.7.1.11	F6P + ATP = FBP + ADP	5.2e3	3.0e4
**Ald**	4.1.2.13	FBP = DHAP + GAP	1.2e-3 mM	1.7e-4 mM
**G3d**	1.1.1.8	DHAP + NADH = G3P + NADH	2.5e7	8.9e6
**Gdh**	1.2.1.12	GAP + NAD + P_i = 13BPG + NADH	9.9e-2 mM^{-1}	9.3e-1 mM^{-1}
**Pgk**	2.7.2.3	13BPG + ADP = 3PG + ATP	2.3e1	2.8e1
**Pgm**	5.4.2.1	3PG = 2PG	9.4e-2	8.7e-2
**Eno**	4.2.1.11	2PG = PEP + H_2O	3.6e0	5.1e0
**Pyk**	2.7.1.40	PEP + ADP = PYR + ATP	6.9e4	3.6e4
**Ldh**	1.1.1.27	PYR + NADH = LAC + NAD	1.5e4	5.4e3

Supplementary Table 23: Thermodynamic equilibria for the enzymatic reactions. Equilibria are given for reactions in direction of glycolysis for ionic strengths of 0 and 0.1M. Deviating from thermodynamic standard conditions, values have been adapted to reactor conditions, specifically pH 7.6 and 1 mM as metabolite concentration for the calculation of Gibbs free energy.

Enzyme	Principle mechanism	Source	Rate law, equation number
**Glk**	Ordered irreversible bi MM kinetics with GLC as first, ATP as second substrate	[1,2]	$r_{\mathrm{GK}} = \frac{V_{\max}[\mathrm{G}][\mathrm{ATP}]}{K_{\mathrm{G}} K_{\mathrm{ATP}} + [\mathrm{G}][\mathrm{ATP}] + K_{\mathrm{ATP}}[\mathrm{G}]}$ (I)
**Glk revised**	Ordered irreversible bi MM kinetics with GLC as first, ATP as second substrate, ADP competitive inhibitor	See main text	$r_{\mathrm{GK}} = \frac{V_{\max}[\mathrm{G}][\mathrm{ATP}]}{K_{\mathrm{G}} K_{\mathrm{ATP}}\left(1+\frac{[ADP]}{K_{\mathrm{i,ADP}}}\right) + [\mathrm{G}][\mathrm{ATP}] + K_{\mathrm{ATP}}\left(1+\frac{[ADP]}{K_{\mathrm{i,ADP}}}\right)[\mathrm{G}]}$ (II)
**Pgi**	Reversible uni-uni MM kinetics	[3]	$r_{\mathrm{PGI}} = \frac{V_{\max}\left([\mathrm{G6P}] - \frac{[\mathrm{F6P}]}{K^{\mathrm{eq}}}\right)}{K_{\mathrm{G6P}} + [\mathrm{G6P}] + \frac{K_{\mathrm{G6P}}}{K_{\mathrm{F6P}}}[\mathrm{F6P}]}$ (III)
**Pfk**	Irreversible ATP MM kinetics with ADP as competitive inhibitor, F6P via cooperative MWC kinetics, PEP as allosteric inhibitor, and A DP as allosteric activator	[4]	$r_{\mathrm{PFK}} = V_{\max} \bar{Y}_{\mathrm{MM}} \bar{Y}_{\mathrm{MWC}}$<br>$\bar{Y}_{\mathrm{MM}} = \frac{[\mathrm{ATP}]}{K_{\mathrm{ATP}}\left(1+[\mathrm{ADP}]/K_{\mathrm{i,ADP}}\right) + [\mathrm{ATP}]}$<br>$\bar{Y}_{\mathrm{MWC}} = \frac{\alpha(1+\alpha)^{n-1} + L\alpha c(1+\alpha c)^{n-1}}{(1+\alpha)^{n} + L(1+\alpha c)^{n}}$<br>$\alpha = \frac{[\mathrm{F6P}]}{K_{\mathrm{F6P}}}$<br>$L' = L_0 \frac{(1+\beta)^n}{(1+\gamma)^n}$<br>$\beta = \frac{[\mathrm{PEP}]}{K_{\mathrm{T,PEP}}}$<br>$\gamma = \frac{[\mathrm{ADP}]}{K_{\mathrm{R,ADP}}}$<br>$c = \frac{K_{\mathrm{R,ADP}}}{K_{\mathrm{T,PEP}}}$ (IV)
**Ald**	Ordered reversible uni-bi MM kinetics with DHAP as first and as GAP second substrate	[5,6]	$r_{\mathrm{ALD}} = \frac{V_{\max}\left([\mathrm{FBP}] - \frac{[\mathrm{GAP}][\mathrm{DHAP}]}{K^{\mathrm{eq}}}\right)}{K_{\mathrm{FBP}} + [\mathrm{FBP}] + \frac{K_{\mathrm{FBP}}}{K_{\mathrm{DHAP}}}[\mathrm{DHAP}] + \frac{K_{\mathrm{FBP}}}{K_{\mathrm{GAP}} K_{\mathrm{DHAP}}}[\mathrm{GAP}][\mathrm{DHAP}]}$ (V)
**G3d**	Irreversible TC kinetics	[7]	$r_{\mathrm{G3D}} = \frac{V_{\max}\left([\mathrm{NADH}][\mathrm{DHAP}]\right)}{K_{\mathrm{NADH}} K_{\mathrm{DHAP}} + [\mathrm{NADH}] K_{\mathrm{DHAP}} + \frac{K_{\mathrm{NADH}} K_{\mathrm{DHAP}}}{K_{\mathrm{NAD}}}[\mathrm{NAD}]}$ (VI)
**Gdh**	Tri-bi MM kinetics with cooperative NAD binding accomodated by Hill coefficient		$r_{\mathrm{GDH}} = \frac{V_{\max}\left([\mathrm{NAD}]^n[\mathrm{GAP}][\mathrm{P_i}] - \frac{[\mathrm{NADH}][\mathrm{BPG}]}{K^{\mathrm{eq}}}\right)}{K^n_{\mathrm{NAD}} K_{\mathrm{GAP}} K_{\mathrm{Pi}}\left(1 + \frac{[\mathrm{NAD}]^n}{K^n_{\mathrm{NAD}}} + \frac{[\mathrm{GAP}]}{K_{\mathrm{GAP}}} + \frac{[\mathrm{P_i}]}{K_{\mathrm{Pi}}} + \frac{[\mathrm{NAD}]^n[\mathrm{GAP}]}{K^n_{\mathrm{NAD}} K_{\mathrm{GAP}}} + \frac{[\mathrm{GAP}][\mathrm{P_i}]}{K_{\mathrm{GAP}} K_{\mathrm{Pi}}} + \frac{[\mathrm{NAD}]^n[\mathrm{P_i}]}{K^n_{\mathrm{NAD}} K_{\mathrm{Pi}}} + \frac{[\mathrm{NAD}]^n \mathrm{GAP}[\mathrm{P_i}]}{K^n_{\mathrm{NAD}} K_{\mathrm{GAP}} K_{Pi}} + \frac{[\mathrm{NADH}]}{K_{\mathrm{NADH}}} + \frac{[\mathrm{BPG}]}{K_{\mathrm{BPG}}} + \frac{[\mathrm{NADH}]\,[\mathrm{BPG}]}{K_{\mathrm{NADH}} K_{\mathrm{BPG}}}\right)}$ (VII)

**Pgk**	Random reversible bi-bi MM kinetics	[8-10]	$$r_{\mathrm{PGK}}=\frac{V_{\max}\left([\mathrm{BPG}][\mathrm{ADP}]-\frac{[\mathrm{3PG}][\mathrm{ATP}]}{K^{\mathrm{eq}}}\right)}{K_{\mathrm{BPG}}K_{\mathrm{ADP}}\left(1+\frac{[\mathrm{BPG}]}{K_{\mathrm{BPG}}}+\frac{[\mathrm{ADP}]}{K_{\mathrm{ADP}}}+\frac{[\mathrm{3PG}]}{K_{\mathrm{3PG}}}+\frac{[\mathrm{ATP}]}{K_{\mathrm{ATP}}}+\frac{[\mathrm{BPG}][\mathrm{ADP}]}{K_{\mathrm{BPG}}K_{\mathrm{ADP}}}+\frac{[\mathrm{3PG}][\mathrm{ATP}]}{K_{\mathrm{3PG}}K_{\mathrm{ATP}}}\right)} \quad \text{(VIII)}$$
**Pgm**	Reversible uni-uni MM kinetics	[11]	$$r_{\mathrm{PGM}}=\frac{V_{\max}\left([\mathrm{3PG}]-\frac{[\mathrm{2PG}]}{K^{\mathrm{eq}}}\right)}{K_{\mathrm{3PG}}+[\mathrm{3PG}]+\frac{K_{\mathrm{3PG}}}{K_{\mathrm{2PG}}}[\mathrm{2PG}]} \quad \text{(IX)}$$
**Eno**	Reversible uni-uni MM kinetics (as water is ubiquitous)	[12,13]	$$r_{\mathrm{ENO}}=\frac{V_{\max}\left([\mathrm{2PG}]-\frac{[\mathrm{PEP}]}{K^{\mathrm{eq}}}\right)}{K_{\mathrm{2PG}}+[\mathrm{2PG}]+\frac{K_{\mathrm{2PG}}}{K_{\mathrm{PEP}}}[\mathrm{PEP}]} \quad \text{(X)}$$
**Pyk**	Modeled as an i rreversible reaction For ADP: MM kinetics, for PEP: cooperative MWC kinetics with ATP as allosteric inhibitior and FBP as allosteric activator	[14-17]	$$r_{\mathrm{PYK}}=V_{\max}\frac{\alpha(1+\alpha)^{n-1}}{L'+(1+\alpha)^{n}}\frac{[\mathrm{ADP}]}{K_{\mathrm{ADP}}+[\mathrm{ADP}]}$$ $$\alpha=\frac{[\mathrm{PEP}]}{K_{\mathrm{R,PEP}}}$$ $$L'=L_0\frac{(1+\beta)^n}{(1+\gamma)^n} \quad \text{(XI)}$$ $$\beta=\frac{[\mathrm{ATP}]}{K_{\mathrm{R,ATP}}}$$ $$\gamma=\frac{[\mathrm{FBP}]}{K_{\mathrm{T,FBP}}}$$
**Ldh**	Reversible TC kinetics	[18]	$$r_{\mathrm{LDH}}=\frac{V_{\max}\left([\mathrm{NADH}][\mathrm{PYR}]-\frac{[\mathrm{PEP}][\mathrm{NAD}]}{K^{\mathrm{eq}}}\right)}{K_{\mathrm{NADH}}K_{\mathrm{PYR}}+[\mathrm{NADH}]K_{\mathrm{PYR}}+\frac{K_{\mathrm{NADH}}K_{\mathrm{PYR}}}{K_{\mathrm{NAD}}}[\mathrm{NAD}]} \quad \text{(XII)}$$

Supplementary Table 24: Enzymatic rate laws for the dynamic model. Abbreviations: MM: Michaelis-Menten, MWC: Monod-Wyman-Changeux (with suffixes T and R indicating the tense and relaxed enzyme state), TC: Theorell-Chance

Supplementary note 1: Rate equations used for the mathematical model

Literature research for *E. coli* glycolytic enzymes was undertaken and rate equations were derived considering known mechanism, sequence of metabolite binding, and effectors. In cases where enzymes were obtained from organisms other than *E. coli*, the literature was consulted for deviations in mechanism and effectors. In general the enzymes from the different host organisms had comparable properties to those from *E. coli*. This indicates that the metabolic regulation of the very basic pathway of glycolysis, mainly by Pfk and Pyk, seems to be conserved within our scope of hosts and application.
Reactions were modeled as irreversible if the calculated thermodynamic equilibria were more than 1000-fold on the product side (in glycolytic direction). For the majority of reactions, rapid equilibrium Michaelis-Menten (MM) type kinetics was applied. In the case of the enzyme Eno (catalyzing a dehy dration reaction) we did not consider water as reactant as in aqueous solutions it is ubiquitous. Enzymes G3d and Ldh, which were shown to use a ping-pong mechanism, were modeled as Theorell-Chance (TC) kinetics. For the two cooperative enzymes with allosteric interactions Pfk and Pyk the Monod-Wyman-Changeux (MWC) model was utilized. As in these cases cooperativity is only towards one of the two substrates, the non-cooperative substrate was modeled as MM kinetics and the rate equation was completed by multiplying the MWC and MM terms.
Special care was taken to include regulatory properties of the pathway members. Complex regulatory mechanisms for the allosteric enzymes Pfk and Pyk are described in the literature. These include for Pfk an allosteric activation by all nucleoside diphosphates (due to the model scope only ADP was considered in the model), competitive inhibition of ADP by ATP, and an allosteric inhibition by PEP [4]. All of these effects were included in the model. For Pyk, these include allosteric inhibition by ATP, GTP, and succinyl-coenzyme A as well as allosteric activation by FBP [14]. Due to the model scope only the interactions of ATP and FBP were integrated into the model. Kinases use the Mg^{2+}-nucleotide complex as enzyme substrate, meaning that MgATP is the real substrate for Glk, Pfk, Pgk and Pyk. Our model does not directly account for this fact, but we prevent the Mg^{2+} concentration from influencing the experimental data by supplementing Mg^{2+} in excess. An overview of the regulation pattern is provided in Fig. 2B.

The reaction mechanisms including sources and the formulated rates laws are shown in Supplementary Table 24.

Supplementary Methods

Abbreviations

Abbreviations of compounds and enzymes are summarized in Supplementary Table 1.

Source of chemicals

Chemicals were obtained from Sigma-Aldrich (Buchs, Switzerland) in the highest available purity unless mentioned otherwise. Labelled GLC [U-$^{13}C_6$] and ATP [U-$^{15}N_5$] were obtained from Cambridge Isotope Laboratories (Tewksbury, MA, USA).

Setup of experimental system

Buffers

Operation of the reaction system required specific buffers that were compatible with enzyme activity and the subsequent analysis by MS. The basic reaction buffer for the experiments in the enzyme membrane reactor consisted of 50 mM NH_4HCO_3, 2.5 mM $MgCl_2$ and 0.4 mM MOPS at pH 7.8.

For enzyme reactions, the reaction buffer was modified where required in order to ensure enzymatic reaction: addition of 1 mM dithiothreitol (DTT) for the stabilization of Gdh and Pgm (as recommended by the suppliers), 1 mM KCl (Pyk requires potassium as a cofactor), and 2 µM 23BPG (the Pgm variant that we used required 23BPG for activation [11]). Where indicated $MgCl_2$ concentration was adapted to ATP concentration to ensure formation of MgATP as substrate for kinases. Depending on the quantification methods (see below) standards were added to this buffer.

After the reaction mix had left the reactor, an MS matrix buffer was used to dilute the reactor effluent in order to condition it for MS-analysis (see Fig. 1A of the main text). This buffer consisted of 5 mM NH_4HCO_3 in a mix of 75% MeOH/25% water (vol/vol). The buffer was adjusted to pH 4.8 by the addition of pure formic acid (typically 5 mL L^{-1}) and degassed before usage. Again depending on which quantification method was applied, standards were added before usage.

Hardware The experimental system consisted of a function generation part, a part in which the enzymatic reaction system was operated, a sample conditioning part, and the final measurement part (Fig. 1A). Function generation was achieved by combining a (multi-channel) HPLC pump and an injection loop. The HPLC pump (Agilent, Santa Clara, CA, USA) was connected through polyether ether ketone (PEEK) HPLC capillaries to the main reactor and operated at a rate of 0.25 mL min^{-1} with reaction buffer and reaction compounds as detailed for the individual experiments. Between pump and reactor we placed an injector with loops of different but defined volumes (varying from 20 to 1,000 µL), thus allowing redirection of the flux from the pump through the loop and flushing the loop content, e.g. enzymes or compounds, into the reactor. Supplying defined compound concentrations in the reaction buffer established a continuous reaction feed; switching abruptly from one to a second reaction buffer allowed implementing step functions. Switching gradually over time allowed implementing a ramp function and even more complex input functions such as a sine can be realized by programming the pump. Using the injector for compounds allowed approximating an impulse by producing a pulse from an injector loop volume small in relation

to the flux provided by the pump. Finally, using it for enzyme supply produced a step function in terms of reactor enzyme concentration.
Reactions were carried out in a continuously operated enzyme membrane reactor (Bioengineering AG, Wald, Switzerland, nominal internal volume of 10 mL) with a custom-manufactured inlay that reduced the volume to approximately half and therefore increased the system dynamics. The vessel was constantly stirred by a magnetic stirrer at 600 rpm and the temperature was maintained at 30°C by flushing the integrated cooling jacket with water from a thermostat. The reactor contained a cellulose triacetate membrane with a cutoff at 20 kDa to retain the enzymes but to let the compounds pass. Bovine serum albumin was injected to a reactor concentration of 1 mg mL^{-1} prior to injecting enzymes for improving enzymatic stability (presumably by passivating critical surfaces, e.g. on the membrane).
The reactor effluent was conditioned for subsequent online MS detection by diluting it between 30- and 100-fold with the MS matrix buffer, supplied via an HPLC PEEK capillary system. Afterwards, the flow was split in such a way that approximately 0.03 mL min^{-1} were directed into the MS. The exact fluxes were calculated from standard compounds as discussed below.

Mass spectrometry

In order to measure compound concentrations in the reactor effluent, mass spectrometry was used. Specifically, a triple quadrupole mass spectrometer (MS) (MDS Sciex 4000 Q-TRAP, Applied Biosystems, CA, USA) with electrospray ionization (ESI) was employed in multiple reaction monitoring (MRM) setting and in negative ion mode. The ESI-MS was generally operated at an electron spray temperature of 200°C, an ion spray voltage of -4,200 V, a channel electron multiplier (CEM) of 2,100 V, and the following gas pressures: curtain gas 30 psi, collision gas 6 psi, ion source gas one 30 psi and ion source gas two 40 psi. The identification of compounds by mass required appropriate settings for the three quadrupoles Q1 to Q3, which were responsible for electron spray ionization, analysis and separation (Q1), collision for further fragmentation (Q2), and fragment analysis (Q3), respectively. The corresponding operating parameters required optimization with respect to signal intensity. For this, the compounds to be analyzed were dissolved to a concentration of 20 µM in 50% methanol/50% water (vol/vol) and injected into the instrument using a syringe pump with a volume flow rate of 30 µL min^{-1}.
First, to initiate the analytic MS-routines, the Q1 spectrum was checked for the presence of molecular ion of the compound to be identified, which was always found to be the strongest signal. On the basis of this ion the instrument manufacturer's characterization optimization routine was started, and the optimization routine scanned through the instrument settings (entrance potential EP, declustering potential DP, collision energy CE, and collision cell exit potential CXP) to identify the optimal parameter set. Results are summarized in Supplementary Table 2. These settings (adapted to the differences in masses between compound and isotopologue) were also applied to labeled compounds.
The routines described above did not allow resolving structural isomers, specifically the pairs G6P/F6P and 2PG/3PG. Instead, we measured and report the sum of concentrations of these pairs in two pools, termed X6P (for G6P/F6P) and XPG (for 2PG/3PG). In an earlier publication, we had reported separate measurements of DHAP and GAP based on a unique molecular ion for DHAP and a persistent negligible concentration of GAP in a phosphate-ion rich background, which tempered the effect of ion suppression [20]. In the present system with a much reduced ion-background, measurements of this DHAP-specific ion did not comply

with our accuracy requirements, and we discontinued this practice. Instead, we determined concentrations of DHAP and GAP as a third pool termed XAP. Finally, there was no standard available for 13BPG, therefore we relied on 23BPG.
In the final measurement protocol the dwell time for measuring a single compound in MRM mode was set to 500 ms resulting in a measurement frequency of 1 Hz for a compound and its standard. Depending on the size of the enzymatic system and the number of compounds the measurement protocol was reduced in order to lower the time for a measurement cycle . The EP can be set only once for one protocol and it was set to -10 V.

General microbiological and molecular biology methods
General microbiology and molecular biology work followed established standard procedures unless otherwise mentioned.

Production of the *E. coli* glucokinase Glk
The *E. coli glk* gene was expressed from plasmid pET30-Glk, a pET-30b(+)-derivative (Novagen) that carries the gene encoding an N-terminally 6xHis-tagged Glk under the control of the T7 RNA polymerase-dependent promoter. Cells were prepared after induction from 1 L of LB medium by centrifugation, resuspended in 10 mL of lysis buffer (50 mM Tris/HCl pH8, 300 mM NaCl, 10 mM imidazol) and lysed in a high pressure homogenizer (Microfluidics M-110P, Westwood, MA) at 4°C and a 1,000 bar pressure drop across the orifice. The clarified supernatant was applied to two self-packed Ni-Sepharose gravity flow columns (2 mL bed volume, GE Healthcare, UK) and eluted with 400 mM imidazol. The eluted protein was dialysed twice against a 200-fold excess of Tris/HCl pH 7.5 containing 1 mM $MgCl_2$. The protein was 95% pure (SDS-PAGE, visual inspection). Aliquots were stored with 10% glycerol at -80˚C. The final enzyme concentration was determined as 4.8 mg mL^{-1} by photometric measurement. The enzyme activity was determined spectrophotometrically by oxidation of glucose 6-phosphate with concomitant reduction of NADP. The specific activity of the Glk preparation was determined as 176 U per mg of purified protein.

Production of cell-free extract (CFX)
For CFX production (used for the synthesis of isotopologues, see section 10 of Supplementary Methods), we constructed the strain *E. coli* BW25113 *ΔfbaB pfkB pykA::kan tpiA::Cm* by phage transductions based on strains from the KEIO-collection [21]. The resulting strain was then used for the production of CFX as described previously [20]. CFX was stored in aliquots at -80°C.

Identification of experimental system

In order to separate the identification of the parameters of the system setup, e.g. volume of the reaction vessel, from the identification of the parameters of the enzymatic system, we determined the transfer function of the experimental system first. The experimental system consisted of the HPLC pump, the injector, the reactor and the dilution device. For this, the system was decomposed into subunits which were identified one after the other (Fig. 1C). A constant flow through the subunit that was to be identified was provided by a syringe pump (for the identification of the injector) or by one channel of the HPLC pump (for all other identification experiments). We applied 1 m M GLC in reaction buffer to generate a perturbation. A step input function was impressed on the system through either switching the HPLC pump to a second channel or to an injection loop with a volume of 1 mL. The response of the system to the step function was recorded by the MS. Neglecting the setup-specific dead times, the injector response was close to an ideal step. For the subsequent identification steps the injector function was taken as an i deal step function. The other system elements generally behaved as first order lag elements (PT_1) with dead time. Again disregarding the dead t ime as a s etup-specific property, their responses were fitted to a continuous stirred-tank reactor (CSTR) model:

$$\frac{dc_{v,\mathrm{GLC}}}{dt} = \frac{F}{V_v}\left(c^{\mathrm{in}}_{v-1,\mathrm{GLC}} - c_{v,\mathrm{GLC}}\right) \tag{1}$$

with F as the rate of the incoming volume flow, V_v as the apparent volume of the device v in question, $c_{v,\mathrm{GLC}}$ as the GLC concentration response in the device v, and $c^{\mathrm{in}}_{v-1,\mathrm{GLC}}$ as the concentration of GLC in the step input or the outlet concentration of the previous device (Fig. 1C). The fitted volumes were

$$\begin{aligned} V_{\mathrm{dilution}} &= 0.162\ \mathrm{mL} \\ V_{\mathrm{pump}} &= 0.286\ \mathrm{mL} \\ V_{\mathrm{reactor}} &= 7.09\ \mathrm{mL} \end{aligned} \tag{2}$$

and Supplementary Figure 1 shows the sensitivity of the model against errors in the identification process by varying the volumes in the simulation by +/- 20% of their identified values. The measured step responses are described very well by the simple CSTR models and the identified volumes are well estimated as the sensitivity analysis shows. In summary, the entire setup was modeled as a system of 3 c oupled and i deally mixed CSTR's as summarized in Supplementary Equation (3).

$$\begin{aligned} \frac{dc_{\mathrm{pump,GLC}}}{dt} &= \frac{F}{V_{\mathrm{pump}}}\left(c_{\mathrm{in,GLC}} - c_{\mathrm{pump,GLC}}\right) \\ \frac{dc_{\mathrm{reactor,GLC}}}{dt} &= \frac{F}{V_{\mathrm{reactor}}}\left(c_{\mathrm{pump,GLC}} - c_{\mathrm{reactor,GLC}}\right) \\ \frac{dc_{\mathrm{dilution,GLC}}}{dt} &= \frac{F}{V_{\mathrm{dilution}}}\left(c_{\mathrm{reactor,GLC}} - c_{\mathrm{dilution,GLC}}\right) \end{aligned} \tag{3}$$

Compound quantification

The signal generated by the mass spectrometer (detected ions over time) (see section Mass spectrometry) had to be linked to the concentration of the specific compound in the reactor. There were two potential main error sources in this process: First ion suppression [22] due to the presence of a variety of different compounds in varying concentrations over time in the reactor and therefore also in the ESI-chamber; and secondly flux instabilities in the passive mixing and splitting steps in the devices after the reactor, where the conditioning of the reactor efflux for MS-analysis relied on a system of thin capillaries and the generated flux pattern depended on pressure drops defined by diameters and lengths of the applied capillaries. In consequence, the flux pattern was sensitive to influences changing the involved pressure drops, such as partial blockage of capillaries by solid particles introduced from the reaction buffer or accumulation of drained enzyme (fragments) or other compounds on the capillary walls. We therefore implemented a set of 3 procedures allowing us to compensate for these effects and to quantify the concentrations of compounds in the reactor based on the MS signals.

First, flux monitoring based on the addition of two different isotopologues of taurine (which is not a part of the reaction system) to the reaction and the MS matrix buffer enabled the determination of the flux ratios post-reactor and in consequence also the dilution over time of the reactor effluent with MS matrix buffer (Supplementary Fig. 2A and B).

Secondly, as ion suppression effects should apply in the same way to isotopologues of the same compound, referencing to isotopologues should lead to accurate measurements of concentrations [23]. Therefore, once the flux ratio was known, the concentrations of a compound in the reactor effluent could be calculated by comparing them to the concentration of its corresponding isotopologue in the MS matrix buffer (Supplementary Fig. 2C).

Thirdly, where an isotopologue was not available, another standard (HEPES) that was not part of the reaction system was added to the MS matrix buffer and metabolite concentrations were calculated from predetermined calibration functions of compounds to the used standard (Supplementary Fig. 2D).

The methods will be described in more detail in the following sections.

Determination of the flux ratio of reaction effluent and MS matrix buffer

The flux ratio was calculated as follows (see Supplementary Fig. 2B): For any compound i the mole flux towards the MS $\dot{n}_{i,\mathrm{MS}}$ can be balanced as the sum of fluxes from the reactor and the MS matrix buffer used for dilution:

$$\dot{n}_{i,\mathrm{MS}} = \dot{n}_{i,\mathrm{reactor}} + \dot{n}_{i,\mathrm{dilution}} \tag{4}$$

The mole flux of compound i is the product of the flow rate F and its concentration c_i, and so Supplementary Equation (4) can be rewritten as

$$F_{\mathrm{MS}} c_{i,\mathrm{MS}} = F_{\mathrm{reactor}} c_{i,\mathrm{reactor}} + F_{\mathrm{dilution}} c_{i,\mathrm{dilution}} \; . \tag{5}$$

Inserting the balance for the flow rates

$$F_{\mathrm{MS}} = F_{\mathrm{reactor}} + F_{\mathrm{dilution}} \tag{6}$$

into Supplementary Equation (5) the concentration of any compound towards the MS can be calculated from

$$c_{i,\mathrm{MS}} = \frac{F_{\mathrm{reactor}}}{F_{\mathrm{reactor}} + F_{\mathrm{dilution}}} c_{i,\mathrm{reactor}} + \frac{F_{\mathrm{dilution}}}{F_{\mathrm{reactor}} + F_{\mathrm{dilution}}} c_{i,\mathrm{dilution}} \quad . \tag{7}$$

Using the dilution factor D_{R} for the dilution of the reactor efflux

$$D_{\mathrm{R}} = \frac{F_{\mathrm{reactor}}}{F_{\mathrm{reactor}} + F_{\mathrm{dilution}}} \tag{8}$$

and D_{D} of the MS matrix buffer flux

$$D_{\mathrm{D}} = \frac{F_{\mathrm{dilution}}}{F_{\mathrm{reactor}} + F_{\mathrm{dilution}}} = 1 - D_{\mathrm{R}} \tag{9}$$

Supplementary Equation (7) can be simplified to

$$c_{i,\mathrm{MS}} = D_{\mathrm{R}} c_{i,\mathrm{reactor}} + D_{D} c_{i,\mathrm{dilution}} \quad . \tag{10}$$

TAU with the standard ^{14}N-isotope was added exclusively to the reactor feed, and s o Supplementary Equation (5) can be written as

$$\dot{n}_{\mathrm{TAU,MS}} = F_{\mathrm{reactor}} c_{\mathrm{TAU,reactor}} \quad . \tag{11}$$

Labelled ^{15}TAU was added exclusively to the dilution feed:

$$\dot{n}_{^{15}\mathrm{TAU,MS}} = F_{\mathrm{dilution}} c_{^{15}\mathrm{TAU,dilution}} \quad . \tag{12}$$

The parameter y_{TAU} is defined as the ratio of the MS-determined signals x for ^{14}N- and ^{15}N-TAU:

$$y_{\mathrm{TAU}} = \frac{x_{\mathrm{TAU}}}{x_{^{15}\mathrm{TAU}}} \tag{13}$$

Assuming that both labeled and unlabeled taurine ionize the same way, y_{TAU} is equal to the ratio of the mole fluxes of labelled and unlabeled taurine into the MS:

$$y_{\mathrm{TAU}} = \frac{x_{\mathrm{TAU}}}{x_{^{15}\mathrm{TAU}}} = \frac{\dot{n}_{\mathrm{TAU,MS}}}{\dot{n}_{^{15}\mathrm{TAU,MS}}} \quad . \tag{14}$$

Using the Supplementary Equations (11) and (12) this can be written as:

$$\frac{\dot{n}_{\text{TAU,MS}}}{\dot{n}_{^{15}\text{TAU,MS}}} = \frac{F_{\text{reactor}} c_{\text{TAU,reactor}}}{F_{\text{dilution}} c_{^{15}\text{TAU,dilution}}} \tag{15}$$

and Supplementary Equation (15) can be combined with Supplementary Equation (13) to the expression

$$y_{\text{TAU}} = \frac{F_{\text{reactor}}}{F_{\text{dilution}}} \frac{c_{\text{TAU,reactor}}}{c_{^{15}\text{TAU,dilution}}} \quad . \tag{16}$$

This equation allows calculating $F_{\text{reactor}} / F_{\text{dilution}}$ from the operator-specified taurine concentrations and the relation y_{TAU} of measured taurine MS ion counts as

$$\frac{F_{\text{reactor}}}{F_{\text{dilution}}} = y_{\text{TAU}} \frac{c_{^{15}\text{TAU,dilution}}}{c_{\text{TAU,reactor}}} \quad . \tag{17}$$

Two assumptions were made in the deriving of this equation: [i] The ionization properties of TAU are not influenced by the heavy N-isotope; and [ii] the only source for unlabeled TAU at the mixing tee is the reactor effluent and the only source for labelled TAU is the MS matrix buffer. Regarding the former assumption, injection of TAU and ^{15}TAU at identical concentrations into the MS via the syringe pump resulted in the same signal intensity (data not shown). Regarding the latter assumption, the certificate of analysis by the supplier for ^{15}TAU stated a pur ity of 99.3% ^{15}N (corresponding to 0.7% ^{14}N). The natural isotope distribution of TAU is known (99.636% ^{14}N and 0.364% ^{15}N). At the targeted dilution rate of 1:100, the TAU mole flux from the reactor is essentially identical to the ^{15}TAU mole flux from the MS matrix buffer, as the TAU flux from the reactor is diluted 100-fold. Hence the isotopological impurities partially compensate each other restricting the error to the difference in purity of 0.336%. In sum it can be said that both assumptions are reasonable.

Concentration determination by reference to isotopologues

As ion suppression effects should apply in the same way to isotopologues of the same compound, referencing to isotopologues should lead to accurate measurements of concentrations [23]. To calculate the reactor concentration of a c ompound using its isotopologues as a reference (Supplementary Fig. 2C), Supplementary Equation (16) can be re-formulated for each compound i whose non-labeled version is present in the reactor effluent and whose heavy isotopologue is present in the MS matrix buffer:

$$y_i = \frac{x_i}{x_{i,\text{labeled}}} = \frac{F_{\text{reactor}}}{F_{\text{dilution}}} \frac{c_{i,\text{reactor}}}{c_{i,\text{labeled,dilution}}} \tag{18}$$

Now the combination of Supplementary Equations (17) and (18) allows calculation of the unknown $c_{i,\text{reactor}}$ on the basis of set concentrations and measured signals by

$$c_{i,\text{reactor}} = \frac{c_{\text{TAU,reactor}}}{c_{^{15}\text{TAU,dilution}}} \frac{y_i}{y_{\text{TAU}}} c_{i,\text{labeled,dilution}} \; . \tag{19}$$

We further analyzed the quantification range for different compounds (GLC, FBP, AMP, ADP, ATP, and the measurement pools of X6P, XAP and XPG), and found the relations to be linear under all relevant conditions over more than 3 orders of magnitude (data not shown).

Concentration determination in reference to a standard in the MS matrix buffer

In order to calculate compound concentrations in the reactor in reference to the HEPES standard (Supplementary Fig. 2D), the ratio z_i of the number of ions per second x_i of compound i detected at the MS and x_{HEPES} of HEPES was taken:

$$z_i = \frac{x_i}{x_{\text{HEPES}}} \; . \tag{20}$$

The relation between metabolite concentration at the instrument $\mathrm{c_{i,MS}}$ and the ratio z_i was taken from a calibration curve that had been obtained as follows: A mix of all relevant compounds at a defined concentration was prepared, distributed over aliquots, and stored at -80°C. Reaction buffer was prepared freshly and diluted 1:100 with MS matrix buffer containing 2 µM HEPES to give the calibration solution. Then an aliquot containing metabolite mix was thawed and diluted 1:100 with calibration solution. This initial metabolite mix was then repeatedly diluted two-fold with calibration solution, so that the metabolite concentration decreased quadratically while the concentrations of the background compounds in the reaction buffer (e.g. $MgCl_2$) and in the MS matrix buffer (HEPES) remained constant. In this, samples corresponding to reactor concentrations between 0.61 nM and 4 mM were prepared. These samples were injected directly into the MS with a syringe pump at a rate of 30 µL min^{-1} which was in the range of typical fluxes of the dilution system to the MS. For each metabolite equation (20) was evaluated at different metabolite concentrations and these data points were used for the identification the parameters a and b of a quadratic model

$$c_{i,\text{MS}} = f(z_i) = a_i z_i^2 + b_i z_i \; . \tag{21}$$

These calibration curves describe the difference in ionization properties between HEPES and the metabolite which, as already suggested by the equation above, was a non-linear relation. For the quantification of metabolite concentrations during experiments, Supplementary Equation (20) was applied to measured time series and concentrations at the MS were calculated from the parameterized calibration function in Supplementary Equation (21). Then, based on Supplementary Equation (10), one finds for a compound originating exclusively from the reactor effluent

$$c_{i,\mathrm{MS}} = D_R c_{i,\mathrm{reactor}} \tag{22}$$

and, with the dilution term defined as in Supplementary Equation (8), the reactor concentration of this compound was calculated from

$$c_{i,\mathrm{reactor}} = \left(1 + \frac{F_{\mathrm{dilution}}}{F_{\mathrm{reactor}}}\right) c_{i,\mathrm{MS}} \ , \tag{23}$$

with the flux ratio from the TAU flux monitoring as given in Supplementary Equation (17).

<u>Consideration of errors</u>

The ^{15}TAU/TAU standard was used in general to observe the flux pattern. While the flux from the MS matrix buffer was constantly maintained at 1 mL min^{-1} by an HPLC pump, the flux from the reactor was prone to variation. During experiments we typically observed dilution rates in the range of 1:25 to 1:100. This supports clearly the idea of measuring relative to a standard that is provided by the MS matrix buffer as its corresponding dilution factor was close to 1 ($0.96 < \mathrm{D_D} < 0.99$, corresponding to flux ratios of reactor effluent to MS matrix buffer of 1:25 to 1:100, see Supplementary Equation (9)). Therefore the maximal difference in concentration at the MS of any compound added to the MS matrix buffer is 3%, which for all practical purposes means the signal is constant. Hence the concentration of a standard, such as HEPES, added to the MS matrix buffer can be used as a reliable reference signal in the measurement system.

By comparing the concentration measurements via the isotopologue standard with those that would have resulted if these concentrations had been evaluated using the HEPES standard for those components for which both methods could be applied, we concluded that the remaining ion-suppression-derived error in the HEPES-method is smaller than 20% for the conditions applied in the experiments described here.

The relation between ionization properties of the standard and the compound of interest are dependent on the instrument. As an example the position and also the wear level of the electrode used in the ESI have an influence on ion production. We considered these effects by renewing calibration curves in a biweekly manner or whenever changes to the instrument were made.

In view of the remaining limitations of the HEPES-based quantification, we limited the use of this method to those situations where no suitable isotopologues were available. This was the case for some of the compounds that were unique to the lower glycolysis, and therefore those experiments that involve sections of the lower glycolysis were evaluated with the HEPES-standard.

Production of isotopologues

As mentioned, we had only heavy isotopologues for ATP and GLC commercially available and therefore proceeded to produce further isotopologues ourselves using CFX in the enzyme membrane reactor setup. The system was provided with reaction buffer containing 2 mM ^{13}GLC, 2 mM ^{15}ATP, 4 mM $MgCl_2$, 2 mM sodium phosphate (Na_2HPO_4 / NaH_2PO_4, adjusted to pH 7.2), 1 mM NAD and 400 µM ^{15}TAU. NAD was provided in unlabeled form. The MS matrix buffer that was used for dilution after the reactor contained 10 µM unlabeled glycolytic compounds and 4 µM TAU for monitoring and quantification purposes. The reaction was started by injection of 7 mg of total protein derived from CFX via the injection loop resulting in a total protein concentration in the reactor of 1 mg mL^{-1}. The reactor was continuously fed with reaction buffer at 0.25 mL min^{-1} for 2.5 h. Once the reactor had reached steady-state, the reactor effluent was directly (without further dilution by MS matrix buffer) collected first on ice and then stored in aliquots at -80°C. In order to account for possible metabolite degradation through freeze and thaw processes in subsequent experiments, a thawed aliquot was always measured against a standard of unlabeled glycolysis compounds and concentrations were determined. The results are summarized below. Please note that labelled NAD and NADH could not be produced in this manner. Furthermore, some compounds of the lower glycolysis (G3P, BPG, PEP, PYR and LAC) could not be produced in sufficiently high concentrations to apply the isotopologue-based quantification method in all experiments (*vide supra*).

Compound: ^{13}GLC; Concentration [mM] in reactor effluent: 0.928; Concentration [µM] used as standard diluted 1:500 in MS matrix buffer: 1.856. ^{13}X6P; 0.076; 0.152. ^{13}FBP; 0.385; 0.770. ^{13}XAP; 0.331; 0.662. ^{3}G3P; 0.021, 0.042; ^{13}BPG; n.d. [a)]; n.d. [a)]. ^{13}XPG; 0.056; 0.112. ^{13}PEP; 0.005; 0.010. ^{13}PEP; 0.005; 0.010. ^{13}PYR; 0.170; 0.340. ^{13}LAC; n.d. [b)]; n.d. [b)]; ^{15}ADP; 0.768; 1.536. ^{15}ATP, 0.955, 1.910. NAD, NADH: n.a. [c)]; n.a. [c)]. ^{15}TAU; 0.395; 0.790.

n.d. not determined; n.a. not applicable

a) Concentration in self-prepared set of labelled compounds remained below or at limit of detection
b) The ^{13}LAC signal was strongly perturbed by an unidentified ion
c) No non-radioactive isotopologues of NAD and NADH were available.

Enzyme stability

The stability of enzymes is a prerequisite for successful experiments and for the validity of the kinetic model which was applied, which is time-invariant in the amount of enzymes. However, enzymes are prone to degradation over time or could be lost through leakage over the membrane in the reactor. We addressed the evaluation of a possible loss in enzyme activity repeatedly in the course of this work. However we did so indirectly. Through whatever underlying effect, a loss of activity would result in the present reaction system in a change of metabolite concentrations over time under steady state conditions. However, we did not notice such system behavior in any of our experiments, which typically did not exceed 120 min after charging the reactor with enzymes. As an example for the long time stability of reactions we refer to the stability in metabolite concentrations in three different reaction systems as shown in Supplementary Fig. 3: first Glk only (panel A), secondly upper glycolysis (including Glk, Pgi, Pfk and Ald, panel B) and thirdly full glycolysis (panel C). We conclude that no significant loss of activity resulted in the experimental system for at least 120 min.

Calculation of thermodynamic equilibria

Reaction equilibria are a function of a variety of influences, such as pH, temperature and ionic strength. In order to decide on the reversibility of enzymatic rate laws and to compute a starting point for the estimation of selected equilibrium constants of the model, we used the biochemical thermodynamics calculator eQuilibrator 1.0 (equilibrator.weizmann.ac.il) [24]. For our reactor conditions we estimated the equilibria for the minimal and maximal value of ionic strength that was deemed applicable. The results are summarized in Supplementary Table 23.

Kinetic model

The kinetic model was constructed by balancing the compounds, formulating rate laws for the enzymes, and embedding them into the identified 3 vessel system of the experimental setup. The kinetic model describes the (essentially glycolytic) reaction network displayed in Fig. 2B on the basis of ODE's. These were derived by balancing the molar fluxes of metabolites within the reactor. Due to the constant volume of the vessel the general form of these balances in terms of concentration is

$$\frac{dc_i}{dt} = \sum_j \alpha_{ij} r_j + D(c_i^{\text{in}} - c_i) . \tag{24}$$

Here, c_i is the concentration of metabolite i, α_{ij} is the stoichiometric coefficient of metabolite i in the j-th reaction with the reaction rate r_j, $D = F/V_{\text{reactor}}$ is the dilution rate in the reactor, and c_i^{in} is the reactor input concentration. A total of 18 compounds were balanced in the reactor as summarized in Supplementary Equation (25).

As the whole system setup – pump, reactor and dilution – consisted of 3 compartments, all compounds were also balanced for the pump and the dilution device according to Supplementary Equation (3), resulting in a system model with 54 ODE's.

Concentration balances

$$\frac{dc_{\mathrm{G}}}{dt}=-r_{\mathrm{Glk}}-D(c_{\mathrm{GLC}}-c_{\mathrm{GLC}}^{\mathrm{in}})$$

$$\frac{dc_{\mathrm{G6P}}}{dt}=r_{\mathrm{Glk}}-r_{\mathrm{Pgi}}-D(c_{\mathrm{G6P}}-c_{\mathrm{G6P}}^{\mathrm{in}})$$

$$\frac{dc_{\mathrm{F6P}}}{dt}=r_{\mathrm{Pgi}}-r_{\mathrm{Pfk}}-D(c_{\mathrm{F6P}}-c_{\mathrm{F6P}}^{\mathrm{in}})$$

$$\frac{dc_{\mathrm{FBP}}}{dt}=r_{\mathrm{Pfk}}-r_{\mathrm{Ald}}-D(c_{\mathrm{FBP}}-c_{\mathrm{FBP}}^{\mathrm{in}})$$

$$\frac{dc_{\mathrm{DHAP}}}{dt}=r_{\mathrm{Ald}}-r_{\mathrm{G3d}}-D(c_{\mathrm{DHAP}}-c_{\mathrm{DHAP}}^{\mathrm{in}})$$

$$\frac{dc_{\mathrm{GAP}}}{dt}=r_{\mathrm{Ald}}-r_{\mathrm{Gdh}}-D(c_{\mathrm{GAP}}-c_{\mathrm{GAP}}^{\mathrm{in}})$$

$$\frac{dc_{\mathrm{13BPG}}}{dt}=r_{\mathrm{Gdh}}-r_{\mathrm{Pgk}}-D(c_{\mathrm{13BPG}}-c_{\mathrm{13BPG}}^{\mathrm{in}})$$

$$\frac{dc_{\mathrm{3PG}}}{dt}=r_{\mathrm{Pgk}}-r_{\mathrm{Pgm}}-D(c_{\mathrm{3PG}}-c_{\mathrm{3PG}}^{\mathrm{in}})$$

$$\frac{dc_{\mathrm{2PG}}}{dt}=r_{\mathrm{Pgm}}-r_{\mathrm{Eno}}-D(c_{\mathrm{2PG}}-c_{\mathrm{2PG}}^{\mathrm{in}})$$

$$\frac{dc_{\mathrm{PEP}}}{dt}=r_{\mathrm{Eno}}-r_{\mathrm{Pyk}}-D(c_{\mathrm{PEP}}-c_{\mathrm{PEP}}^{\mathrm{in}})$$

$$\frac{dc_{\mathrm{PYR}}}{dt}=r_{\mathrm{Pyk}}-r_{\mathrm{Ldh}}-D(c_{\mathrm{PYR}}-c_{\mathrm{PEP}}^{\mathrm{in}})$$

$$\frac{dc_{\mathrm{LAC}}}{dt}=r_{\mathrm{Ldh}}-D(c_{\mathrm{LAC}}-c_{\mathrm{LAC}}^{\mathrm{in}})$$

$$\frac{dc_{\mathrm{G3P}}}{dt}=r_{\mathrm{G3d}}-D(c_{\mathrm{G3P}}-c_{\mathrm{G3P}}^{\mathrm{in}})$$

$$\frac{dc_{\mathrm{ATP}}}{dt}=-r_{\mathrm{Glk}}-r_{\mathrm{Pfk}}+r_{\mathrm{Pgk}}+r_{\mathrm{Pyk}}-D(c_{\mathrm{ATP}}-c_{\mathrm{ATP}}^{\mathrm{in}})$$

$$\frac{dc_{\mathrm{ADP}}}{dt}=r_{\mathrm{Glk}}+r_{\mathrm{Pfk}}-r_{\mathrm{Pgk}}-r_{\mathrm{Pyk}}-D(c_{\mathrm{ADP}}-c_{\mathrm{ADP}}^{\mathrm{in}})$$

$$\frac{dc_{\mathrm{Pi}}}{dt}=-r_{\mathrm{Gdh}}-D(c_{\mathrm{Pi}}-c_{\mathrm{Pi}}^{\mathrm{in}})$$

$$\frac{dc_{\mathrm{NADH}}}{dt}=r_{\mathrm{Gdh}}-r_{\mathrm{Ldh}}-r_{\mathrm{G3d}}-D(c_{\mathrm{NADH}}-c_{\mathrm{NADH}}^{\mathrm{in}})$$

$$\frac{dc_{\mathrm{NAD}}}{dt}=-r_{\mathrm{Gdh}}+r_{\mathrm{Ldh}}+r_{\mathrm{G3d}}-D(c_{\mathrm{NAD}}-c_{\mathrm{NAD}}^{\mathrm{in}}) \tag{25}$$

Optimization

In order to search the parameter space for parameter estimation and optimizing enzyme concentrations a Gaussian adaptation algorithm [25] served as optimizer. The used algorithm is a probabilistic method resulting in different outcomes for different optimization runs and so by default multiple runs were performed to ensure optimal results.

For the estimation of parameters p, the mean squared error function between measurement and simulation was minimized as a quality criterion:

$$\min_{\mathrm{p}} \frac{1}{n}\sqrt{\sum_{i=1}^{n}\left(c_{\mathrm{m}}\left(t_i\right)-c_{\mathrm{s}}\left(t_i,\mathrm{p}\right)\right)^2} \tag{26}$$

with c_{m} representing the measured and c_{s} the simulated concentrations at time point i for all n points of observation. For the optimization of enzyme amounts E, the objective function was the maximization of specific final metabolite concentration $\mathrm{c_i}$ at the simulation end time t_{end} defined by the user

$$\max_{\mathrm{E}} \sum_{\mathrm{i}} \mathrm{c_i}(\mathrm{E}, \mathrm{t_{end}}) \ . \tag{27}$$

From a practical perspective we wanted to optimize the distribution of a given amount of total enzyme $\mathrm{E_0}$ (in units) resulting in a constrained optimization problem with

$$\mathrm{E_0} = \sum_{\mathrm{i}} \mathrm{E_i} \tag{28}$$

as the constraint. We realized this by optimizing the proportions $\mathrm{k_i}$ of enzymes instead of directly optimizing the enzyme amounts $\mathrm{E_i}$ to fulfill the conservation of Supplementary Equation (28). For example, for a two enzyme system the relation between the first and the second enzyme would be formulated as

$$\mathrm{E_1} = \mathrm{k_1 E_2} \tag{29}$$

and the conservation from Supplementary Equation (28) would be

$$\mathrm{E_0} = \mathrm{E_1} + \mathrm{E_2} \ . \tag{30}$$

In the optimization only the proportion $\mathrm{k_1}$ would be adapted while inserting Supplementary Equation (29) into (30) allows the calculation of the last, here second, enzyme amount:

$$\mathrm{E_2} = \frac{\mathrm{E_0}}{1-\mathrm{k_1}} \tag{31}$$

This process can be expanded for n enzymes in n-1 calculations.

Data processing and error model

In the section “Compound quantification” we presented the strategies and methods for the conversion of MS signals into concentrations in the reactor. The practical implementation of these strategies required a work-flow that was able to deal with the imperfection of real data. Different types of errors like crosstalk between compounds, offsets in the measurements and errors in quantification were addressed in the form of a pipeline - a fixed sequence of steps to treat data consistently. The general workflow of the data processing pipeline is illustrated in Supplementary Fig. 13 and included the following steps:

Elimination of crosstalk in raw data

First of all we corrected the raw data from crosstalk between adenosine nucleotides. Injection of ATP of the highest purity available commercially (≥ 99% according to the certificate of analysis) into the MS resulted in signals for ADP and AMP that could not be solely explained by the remaining impurities in the ATP preparation. We therefore assume that this phenomenon is a consequence of the electron spray ionization process, during which one or two phosphate groups could be lost from ATP, leading to the *in situ* creation of two other compounds, one of which is part of the experimental measurement.

During experiments, the ATP-signal from the feed-only phase (before the injection of the enzyme(s)) was used to calibrate this percentage anew for each experiment. In the following experiment, the ADP-signal was then corrected as a function of the ATP signal according to this calibration. We did not observe similar behavior for any other metabolite.

Calculation of metabolite concentrations

The metabolite concentrations in the reactor were calculated according to the different quantification methods as described earlier. These raw concentration time series were stored as data set "c1" for further processing (see also Supplementary Fig. 12 at the bottom of this section).

Matching the observed and set concentrations

Where these calculations led to non-closing mass balances during those times where the system was under complete control (e.g. before the addition of enzymes to start the reaction), these differences were used for corrections, leading to data set “c2”. Specifically, signals of compounds that were sure to be absent in the feed at the start of the reaction were taken as offsets and subtracted from calculated concentrations. These typically low errors can be tracked to different types of contamination: remaining quantities within the experimental system (in particular the post-reactor tubing), pollution of the MS and impurities of used compounds.

For compounds with initial experimental concentrations larger than zero, the ratio between the set and the measured concentration

$$k = \frac{c_1(0)^{\text{set}}}{c_1(0)^{\text{measured}}} \tag{32}$$

was used as scaling factor to correct the complete time series

$$c_2(t) = kc_1(t) \quad . \tag{33}$$

Possible reasons for these differences for compounds with initial concentrations larger than 0 have to be distinguished according to the quantification method:
For the HEPES standard based quantification method, ion suppression by different ion backgrounds is the most likely source of error. In agreement with this, the observed error in the compound balance was typically much lower for the isotopologue standard. This presumable error source suggested that a scaling factor was a more suitable correction than a constant addition or subtraction as applied above.
However, even for the isotopologue standard the error was not always negligible. Possible reasons included instrument tuning-specific limitations with the exact distinction between TAU and ^{15}TAU at low dilution rates in the post-reactor tubing section (i.e., when dilution deviated substantially from the 1:100 target value, the two signals might overlap in the MRM analysis) and pos sible errors in the quantification of the labelled standard itself, which needed to be repeated regularly and showed distinct variations as a function of the continuous use of the MS. Furthermore, for economic reasons we used the isotopologues in the corresponding standard in the lowest possible concentration, which allowed a good and reliable quantification of the reactor-concentrations in most scenarios, but was susceptible to contaminations in some situations, the reasons for which were not always possible to discern.
For experiments involving only the enzymes of upper glycolysis with Ald as the last enzyme of the reaction system, the concentrations of DHAP and GAP were obtained by dividing the measured concentration pool XAP into two equal parts as both compounds are produced with identical stoichiometry by Ald.

Calculation of compound balances

In order to further improve data consistency of the observed concentrations we took into account the conservation of species, which had to be computed. For the most general case - the full reaction system - four conserved moieties were selected, reflected in the carbon-balance, the NAD(H)-balance, the AXP-balance and the P_i-balance.
The carbon balance can be written down in different ways, e.g. taking either compounds with 3 carbon atoms (C3) or with 6 carbon atoms (C6) as basis, but these can be transformed into each other by a factor. Written for C6, the carbon balance can be formulated as

$$\begin{aligned}\sum_{C6} &= [GLC] + [X6P] + [FBP] \\ &\quad + 0.5\big([XAP] + [G3P] + [13BPG] + [XPG] + [PEP] + [PYR] + [LAC]\big)\end{aligned} \quad . \tag{34}$$

The NAD(H) balance can be formulated as

$$\sum_{N} = [NAD] + [NADH] \quad . \tag{35}$$

The AXP balance

$$\sum_{A} = [ATP] + [ADP] \quad (36)$$

can be formulated intuitively while the phosphate balance P_i is more complicated as it follows the phosphate group(s) and some compounds can be phosphorylated twice, resulting in

$$\sum_{P_i} = [ATP] + [X6P] + 2[FBP] + [XAP] + [P_i] + 2[13BPG] + [XPG] + [PEP] + [G3P] \ . \quad (37)$$

Although these 4 formulated balances hold true generally, not all balances were helpful or meaningful in each scenario. We practically operated with 3 different scenarios, namely upper, lower and full glycolysis. For upper glycolysis, the NAD(H) balance does not play a role as neither NAD nor NADH is part of the system, but the C, the AXP, and the P_i-balance could be used. For the lower and the full glycolysis, NAD(H), C, and AXP-balance could be used while the P_i-balance was obsolete due to not measureable P_i. For experiments with a constant feed and without perturbations, the balances are constant over time. However, this is not true for other perturbation scenarios, for which the total amount of compounds in the system could change over time. For dynamic feed profiles and injections, the feed profiles were taken into account to compute the theoretical total compound concentrations in the system in the reactor for each time point of the experiment allowing calculating the balances over time.

Matching of ideal and real mass balances

In the previous section we described the calculation of ideal $\sum_{ideal}(t)$ mass balances by simulation and real $\sum_{real}(t)$ mass balances from experimental data. Based on these a time dependent correction factor $k(t)$ can be determined as

$$k(t) = \frac{\sum_{ideal}(t)}{\sum_{real}(t)} \ . \quad (38)$$

This correction factor was then used to correct the c2 concentration series

$$c_{3/4}(t) = k(t)\, c_2(t) \ . \quad (39)$$

This means essentially that the error in the mass balance was distributed equally over the considered concentrations in order to force the concentration balance to close. Certainly the assumption that the observed error is caused by all compounds to the same extent is not necessarily true, but it seemed the least biased way to handle unknown error sources.

In choosing which concentrations were to be corrected according to equation (39) we followed two paths: in the first scenario (leading to the data set "c3") we considered the already performed corrections described in “Matching the observed and set concentrations” for compounds with an initial concentration larger than zero as sufficient and refrained from correcting them again. Hence the remaining error was distributed only over those compounds with initial values equal to zero. In the second scenario (data set "c4") all

compounds of the balance were corrected regardless of previous corrections. In consequence the difference lies only in the few substrates provided with the initial feed, e.g. GLC and ATP. Both ways are also shown in the Supplementary Fig. 13.
While the usage of the correction factor is straight forward, one aspect had to be considered: the presented balances are only partially interdependent as they share compounds through the phosphate balance. For example FBP is part of the carbon (see Supplementary Equation (34)) as well as of the phosphate balance (Supplementary Equation (37)) and ATP is part of the adenosine (Supplementary Equation (36)) and of the phosphate balance (Supplementary Equation (37)). In consequence, if a balance was used to correct certain concentrations this influenced the remaining balances and therefore a certain order in applying the balances had to be chosen. This was only necessary for the upper glycolysis as only there the phosphate balance was used. We applied the balances in the sequence of carbon, phosphate, and finally AXP balance, which in our hands delivered the best results.
For the lower and full glycolysis the phosphate balance could not be evaluated as the phosphate was not part of the measured compounds and the remaining balances did not overlap in compounds, so no ranking was required.
Injecting the diluted compound mix from the section “Concentration determination in reference to a standard in the MS matrix buffer” into the MS showed for the compounds 13BPG and ATP a retained signal in time reaching steady-state later than the other compounds. At the same time, when the injection was stopped and the system was flushed by pure buffer again a time delayed response giving a signal trail was observed. This effect, reminiscent of chromatography, is most likely the result of binding/unbinding interactions between the PEEK tubing and these compounds. While 13BPG concentrations were experimentally very small and the compound was not used in the feed (commercially unavailable), consequences of the time delay for the widely used ADP/ATP cofactors were of concern. Together with the data processing, which uses initial concentrations as reference where possible, a delay in signal establishment at the beginning could result in overcorrecting this compound, which also could influence the correction of the concentrations of other compounds via the closing of mass balances. At the start of an experiment only ATP was present but no ADP, hence the only compound responsible for a possibly wrongly determined initial concentration was ATP which could be corrected. Later, when due to reaction ATP and ADP are present, both compounds may contribute to a measurement error. As we did not know which compound contributed how strongly to the error we decided to distribute the error to both compounds relative to their actual values. If this measure is wrong we would have corrected over time with an increasing error and this could explain why the deviations in ADP/ATP concentrations were only observed in parts of the data. We did not observe this for the other compounds. This is also the reason why the ADP/ATP cofactors pair was not fitted and therefore not used for parameter estimation.

Parameterization

Boundary conditions for parameterization

For the initial parameterization of the reaction model and the definition of parameter boundaries for the parameter estimation we used different sources. V_{max} values were obtained from the known amount of added enzymes, equilibrium constants were calculated from thermodynamic data and intrinsic enzymatic properties such as K_M were obtained from databases such as BRENDA [19] and literature research.

Hereby the following considerations applied to the process of parameterization of the reaction system: Generally the upper and lower boundaries in which parameters were allowed to vary during estimation were set following fixed rules: providing 1 U of activity in the reactor would translate into a V_{max} 0.143 mM min^{-1}. The activity definition of most enzymes of lower glycolysis by the supplier is valid for 25°C (see Supplementary Table 3) while the reactor temperature was set to 30°C. Using the logarithmic Arrhenius relationship for the temperature dependency of reactions as a first estimate for the temperature-dependent change of enzymatic activity suggested an activity of 0.230 mM min^{-1}. Therefore, we fixed boundaries for V_{max} values between 60 and 250% of this temperature-adapted reference activity, from 0.143 to 0.572 mM min^{-1} in terms of activity in the reactor.

Because of frequently broadly varying literature values, we attributed the lowest certainty to parameters describing enzyme-compound interactions, and consequently the estimation of affinities or K_M values was constrained only by what were considered reasonable physiological ranges, e.g. between 1 µM and 2 mM. This also allowed testing the ability of our procedures to perform effectively *de novo* parameter estimation, as in many similar scenarios which do not rely on well-known enzymes such as those derived from glycolysis, little enzyme data will be available from the literature. In contrast, V_{max}-values (as the product of k_{cat} and enzyme concentration), thermodynamic equlibria, and Hill coefficients, were considered to be of a more certain character and for them the estimation process was constrained to more narrow ranges, e.g. between 0.5 and 2 times the initial value for V_{max} and thermodynamic equilibria while Hill coefficients were constrained around the number of reported enzyme subunits, e.g. between 3 and 4 for a tetrameric enzyme.

Parameterization of catalysts for pool reactions

As discussed, certain reactions in upper glycolysis could not be fully identified with the applied MS-system: Pgi, Ald and Pgm. Consequently, the corresponding concentration information was only available as a pool. Hence the parameter values for the enzyme rate equation could not be identified from the experiments. This problem was addressed as follows:

Pgi (G6P = F6P, [G6P] + [F6P] = [X6P]): Values for the affinities were taken from literature [3] and as equilibrium constant we used the calculated value from our thermodynamics calculations which was constant for the assessed range of conditions.

Ald (FBP = GAP+DHAP, [GAP] + [DHAP] = [XAP]): As the substrate FBP and the XAP pool were distinguishable and the stoichiometry of the reaction was pre-defined, the problem of not sufficiently different MS-signals for GAP and DHAP did not limit the parameterization. However, during preparatory estimation runs we observed that the measured concentrations of DHAP and GAP could not be reproduced with the calculated Ald equilibrium even if widely open parameter boundaries were selected for the affinities. One simple explanation for such

a systematic error is an error in the determination of the equilibrium constant of the Ald-catalyzed reaction. The value obtained from the formalism used to calculate the equilibrium constants was extremely low, which seemed to be inconsistent with the naturally required reaction rates through this enzyme, pointing indeed to a systematic error in the computation of the equilibrium constant. This constant is derived from literature adapted to user-specified modifications in the experimental conditions (see section "Calculation of thermodynamic equilibria") and the measurement of enthalpy changes. However, the determination of the equilibrium constant of Ald is known to be error prone [26]. Therefore, we used the experimentally observed concentrations of FBP and the XAPs to calculate the XAP/FBP equilibrium as 0.036 mM (instead of 1.2e-3 to 1.7e-4 mM as calculated above). This value was consistent with values theoretically calculated and used in other models (e.g. [27,28]) and we used it as a constant in the model together with the affinities from literature [29].

Pgm (3PG = 2PG, [3PG] + [2PG] = [XPG]): In analogy to the Pgi situation we started with constant literature values for Pgm, but could not reproduce the experimental results satisfactorily. In consequence we started estimating the parameters *de novo*, too. The concentration time series could be reproduced well by various estimated parameter sets which was expected due to the inherent non-identifiability caused by the non-observability. Consequently we reduced the parameter boundaries to reasonable ranges obtained from literature reported values [19] and were able to fit the experimental data within these boundaries.

Parameterization of subsystems in general

The model of the enzymatic reactions has 60 parameters whose values have to be identified by computationally fitting experimental time series. Given the available computation power and the parameter space to be searched (which grows exponentially with each parameter), the estimation of all parameters at the same time was not feasible. Hence the overall problem was split into manageable sub-problems of maximally 4 enzymes of around 20 parameters as shown in Supplementary Fig. 5. The breakdown of the system was structured according to the availability of substrates, the observability of compounds, and the requirement that the thermodynamic equilibria of reactions should allow a sufficient extent of reaction.

Subsystem A

The 4 parameter system was elaborated as an example for structural identification. It contained only Glk, whose few parameters allowed manually investigating results and test procedures, in the course of which we identified and corrected an inconsistency between the rate equation reported in the literature and our results.

Subsystem B

The system size was increased to upper glycolysis from Glk to Ald, already producing DHAP (22 parameters).

Subsystem C

The 23 parameters choice was influenced by two considerations: firstly GAP was not a good starting point as it was not commercially available in suitable purity, implying that FBP and Ald were needed as part of C to produce GAP. This implied also the production of DHAP which then could be consumed by G3d. Secondly, thermodynamic calculations suggested that the equilibrium of the Gdh reaction is strongly on the GAP side and so to observe appreciable reaction the product 13BPG must be removed by the action of Pgk.

Subsystem D

The composition of subsystem D (8 parameters) was motivated by the fact that 2PG was again unavailable at suitable purity. Further, the Pgm-catalzyed reaction could not be observed as substrate and product and thus could not be distinguished by MS. However, 2PG could be transformed by Eno into PEP, which could be observed.

Subsystem E

Both, Pyk- and Ldh-catalyzed reactions are strongly on the side of the products and were identified together in subsystem E (12 parameters).

Subsystem F

Reconciliation of the various subsystems into one single model required re-estimation of some parameters. The two NADH consuming reactions Ldh and G3d had to be estimated again together as subsystem F (16 parameters). Pyk was included to provide consistency.

Subsystem G

Furthermore, Pfk and Ald were re-estimated as subsystem G (11 parameters) integrating data from reduced and full reaction system to maintain consistency.

Parameterization of subsystem A (Glk only)

We first applied different input functions (Fig. 2A and Supplementary Fig. 4A, column a, also Supplementary Table 4) to obtain a parameter estimate for purified *E. coli* Glk using a rate equation derived from literature [1]. As this was the first subsystem we investigated, we looked for each type of experiment at the optical fit quality (Supplementary Fig. 4A, column b), the distribution of the achieved errors (Supplementary Fig. 4A, column c), and the distribution of parameters allowing the representation of the experimental data within 1% of the minimal error (Supplementary Fig. 4A, column d). Clearly, it was impossible to obtain with any of the different input functions a narrow distribution of all the parameters for runs that fulfilled the 1% criterion (Fig. 2A, upper panel and Supplementary Fig. 4A, column d). This indicated a problem of practical non-identifiability for this combination of experiment and model.

Next, we incorporated a product inhibition by ADP into the model rate equation for the Glk reaction (in the form of an apparent affinity K_{ATP}^{app}) (see also Supplementary Note 1, Supplementary equations (I) and (II)) and repeated the analysis (Fig. S4B). The experiments had again very good optical qualities of fit, similar to the situation for the enzyme mechanism without inhibition (data not shown). The quality of fit distributions for all types of experiments also remained comparable (Supplementary Fig. 4A and B, column c). The best qualities of fit for type I (constant) and II (step) experiment stayed almost constant irrespective of which mechanism was applied for Glk and similar problems with respect to the affinities remained, suggesting that the non-identifiability problem persisted for these experiments. However, the quality of the fittings for the type III (ramp) experiment was markedly improved and delivered a unique minimum which could be assigned to a single parameter set with very good accuracy (Fig. 2A, lower panel, Supplementary Fig. 4B, column d, and Supplementary Table 5).

Finally, as a control, we conducted an experimental confirmation for an inhibition of Glk by ADP (Supplementary Fig. 4C) by running constant feed experiments, with GLC or G6P and with GLC and ADP in the feed. We placed 1 U of Glk in the reactor and tracked the concentration of GLC.

Parameterization of subsystem B

Experiments were planned to rationally target different aspects (i.e., parameters) of subsystem B with different types of experiments, specifically constant feed experiments and pulse experiments (Supplementary Table 6). Pulse experiments were expected to allow improved estimation of affinities: Tailored injection of compounds allowed the setting of individual compound concentrations independently from the reaction system by deflecting it from the steady-state of substrate and product concentrations. The dynamic response of (constant) enzyme to changing compound concentrations would then reflect the interactions between enzyme and metabolites (affinities). In the first pulse experiment (B4 in Supplementary Table 6) we sequentially pulsed metabolites of upper glycolysis after an initial steady-state had established (Supplementary Fig. 6). In experiment B5 we exposed the cooperative Pfk to pulses in known allosteric effectors, namely the inhibitor PEP and the activator ADP with a second role as competitive inhibitor to ATP, and in B6 we screened for effectors of upper glycolysis by sequentially pulsing metabolites of lower glycolysis. However, we did not observe such effects besides the already known inhibition of Pfk by PEP (Supplementary Fig. 6F). The resulting set of parameters for the enzymes from Glk to Ald is summarized in Supplementary Table 7.

Parameterization of subsystem C

Please note that for the estimation of parameters for subsystems C to E, we had one experiment (E5) that was used – together with other experiments - for the estimation of all three subsystems. In this experiment, the lower glycolysis was built up stepwise by subsequently injecting single enzymes once the injection of the previous enzyme had resulted again in a steady state with respect to the metabolite concentrations. This generated an experiment that contained all reactions of lower glycolysis. From this experiment, three individual data sets C3, D3 and E5 were obtained by considering different end times.

The subsystem C contains a large number of parameters for 4 reactions and is interdependent as the G3d reaction requires NADH formed by the Gdh reaction. Furthermore the predicted equilibria and also the experimental observations showed that the equilibrium of the Gdh reaction could be expected to be on the substrate side and thus efficient flux through this enzyme required removal of the reaction product 13BPG by Pgk. Thus we performed a two-step approach in experiments and in parameter estimation. First, we used experiments C1 to C3 (Supplementary Table 8, Supplementary Fig. 7) to estimate the parameters for Ald, Gdh and Pgk. Next, we used experiments C1 and C4 to estimate the parameters of G3d and performed simultaneously a re-estimation of the parameters of Gdh while keeping the parameters of Ald and Pgk constant. As both enzymes Gdh and G3d act on the NAD/NADH cofactors this allowed (correctly) balancing both reactions and at the same time reducing the numbers of parameters to be estimated. This delivered the parameter set summarized in Supplementary Table 9 which was used for the simulations in Supplementary Fig. 7.

Parameterization of subsystem D

Compounds 3PG and 2PG were only measured as a pool (XPG), impeding the experimental observability of the Pgm reaction. All three compounds were commercially available but 2PG only in low purity of around 70%. As we suspected 3PG to be the most important impurity in the commercial preparation, we abstained in the experiments from using 2PG as we did not want to work with undefined feeds or pulses. Hence the experiments D1 and D2 (Supplementary Table 10, Supplementary Fig. S8) started with 3PG as feed substrate in a concentration typically observed by us in glycolysis experiments. During the fitting process we observed that the experiments could be fitted exceptionally well within the broad *de novo* boundaries, such as K_M values between 1 µM and 2 mM, but 3 of the 4 K_M's were at the lower boundary. This outcome seemed to be rather unrealistic as when compared with the literature [19]. Therefore, we selected boundaries more in the physiological range (Supplementary Table 11). Please note that only one of three concentrations in subsystem D could be observed directly limiting the identifiability of parameters as e.g. high 2PG concentrations can be compensated by low affinity of an enzyme for 2PG.

Parameterization of subsystem E

Subsystem E contained 12 parameters and was estimated using the experiments E1 to E5 (Supplementary Table 12, Supplementary Fig. 9) to obtain the parameter set of Supplementary Table 13. While the fitted concentrations are generally in good agreement, the cofactors ADP, ATP, NAD and NADH qualitatively reproduce the measurements but show slight offsets.

Parameterization of subsystem F

The previous parameter estimations for the subsystems C to E had led to a fully parameterized model of the enzymatic reaction system of lower glycolysis which we had verified for the production of LAC using Ldh (Supplementary Fig. 9). As an additional control we also evaluated it against experimental data of the all lower glycolysis enzymes including G3d for the production of G3P. In this control we observed a mismatch of the model (data not shown), suggesting that the parameters for both enzymes needed to be estimated in the same experiment. To assure consistency with previous results and data bases we re-estimated subsystem E together with G3d resulting in the subsystem F. We used again the data from experiments E1 to E5 and added experiments F1 and F2 (Supplementary Table 14, Supplementary Fig. 10). From these 7 experiments the 13 parameters for the enzymes Pyk, Ldh and G 3d were estimated by fitting the concentrations of DHAP, G3P, PYR and LAC. The estimated parameter values are given in Supplementary Table 15.

Parameterization of subsystem G and verification experiments for full glycolysis

As control and reference for the complete reaction system from GLC to either PYR/G3P or LAC we set up 2 experiments using 1 U of every enzyme and either relying on Ldh or G3d for the NAD regeneration (experiments G1 and G2 in Supplementary Table 16). Here, we operated for the first time the full reaction system from GLC to the products LAC or G3P, respectively, and we observed a major discrepancy in the FBP concentration (data not shown). As a result, we decided to re-estimate the parameters of Pfk and Ald again by fitting them to the concentrations of GLC, X6P and FBP, using (for consistency reasons) the experiments B1 to B6 and G1 and G 2. Newly estimated were the parameters maximal reaction velocities, affinities and equilibria while other parameters of Pfk, e.g. the Hill coefficient, were kept constant at their previously identified values. The resulting set of parameters for Pfk and Ald is summarized in Supplementary Table 17 and the simulations of the experiments G1 and G2 using this data set are displayed in Supplementary Fig. 11. With this final parameter adaption we had developed a fully parameterized model of the reaction system being able to describe the reactions from Glk to Ldh. The corresponding overall parameter set is summarized in Supplementary Table 18.

Optimized reaction system

The optimization of the reaction system was performed by adapting the activity of single enzymes for a given total amount of activity with regards to one or two compound concentrations. For this the optimizer suggested an activity distribution and simulated the experimental outcome of a constant feed experiment with initial enzyme injection for a chosen experimental length. The finally reached concentration at steady-state was taken as criterion and provided to the optimizer that then suggested another distribution. As starting point for the optimization an equi-activity scenario with every enzyme having the same specific activity was chosen. As parameters for the enzymatic reaction the actually available best parameter set from the stepwise identification of the reaction system was used.

Optimization of upper part of reaction system

With the parameter set of Supplementary Table 7, which had been identified for the upper part of the reaction system, we proceeded to optimize system performance of this section. Specifically, we wanted to optimize the distribution of a given amount of enzyme activities (given total number of units enzyme supplied) to obtain maximum product formation, here specifically for DHAP. DHAP is rapidly produced by Ald in an equilibrium-limited reaction from FBP, which in turn is formed in an irreversible reaction from F6P. Therefore, in conceptual terms, the relevant optimization problem is the formation of FBP, from which DHAP can simply be formed by addition of sufficient Ald to allow rapid establishment of equilibrium. In consequence we optimized FBP formation by distributing a total of 3 U of enzyme activity over Glk, Pgi and Pfk. We optimized for the experimental conditions of constant feed experiments (2 mM GLC and 2 mM ATP) and an experimental time of 60 min, in which the system reached steady-state. The fact that we used non-stoichiometric amounts of ATP (2 moles of ATP are needed to convert 1 mole of GLC to FBP) was of no particular concern, as ultimately ATP was expected to be regenerated in the entire reaction system by including the lower part of glycolysis. Optimization results as shown in Supplementary Table 19 and Figure 3A.

Optimization of lower part of cascade

The lower reaction cascade with NAD regeneration by G3d was optimized for a constant feed experiment (1 mM FBP, 2 mM ADP, 1 mM NAD and 2 mM P_i) and maximum G3P production as the objective. The total amount of activity for the seven-enzyme system was 7 U. Parameters for Ald, Gdh and Pgk were extracted from Supplementary Table 9, for Pgm and Eno from Supplementary Table 11 and for Pyk, Ldh and G3d from Supplementary Table 15. The designed distribution of enzyme activity is shown in Supplementary Table 20 and the behavior of the reaction system in Fig. 3B.

Optimization of entire cascade

The whole cascade, with G3d instead of Ldh, was optimized for the concentrations of G3P as end product and PYR for ATP regeneration for a 60 min constant feed experiment (2 mM GLC, 2 mM ATP, 1 mM NAD and 2 mM P_i). As parameters we used the final parameter set from Supplementary Table 18. Two experiments were designed for 20 and 40 U of total enzymatic activity and results are shown in Supplementary Table 21 and Fig. 3C.

Minimization of NAD concentration in the feed

As a further application of the model, we investigated to what extent the NAD concentration in the feed could be reduced without affecting the performance of the reaction system. Although NAD concentration was already sub-stoichiometric in the scenarios investigated so far, we were interested in further decreasing the NAD requirement as it is by far the most expensive feed compound. We used the model with the final parameter set from Supplementary Table 18 to simulate the effect of decreasing NAD concentrations on the performance of an equi-activity distributed system (1 U of every enzyme) with constant feeds (2 mM GLC, 2 mM ATP and 2 mM P_i), which contained different concentrations of NAD, ranging from 0.1 mM to 2 mM. Simulation results are given in Supplementary Fig. 12. Then, we optimized the distribution of enzymatic activities for the above described reaction conditions and 0.25 mM NAD for a 60 min continuous feed experiment (concentration as

above) with regards to final G3P concentration. The optimization results are summarized in Supplementary Table 22 and shown in Fig. 3D.

Supplementary References

1 Lunin, V. V. *et al.* Crystal structures of *Escherichia coli* ATP-dependent glucokinase and its complex with glucose. *J. Bacteriol.* **186**, 6915-6927 (2004).

2 Meyer, D., Schneider-Fresenius, C., Horlacher, R., Peist, R. & Boos, W. Molecular characterization of glucokinase from *Escherichia coli* K-12. *J. Bacteriol.* **179**, 1298-1306 (1997).

3 Schreyer, R. & Bock, A. Phosphoglucose isomerase from *Escherichia coli* K 10: purification, properties and formation under aerobic and anaerobic condition. *Arch. Microbiol.* **127**, 289-298 (1980).

4 Blangy, D., Buc, H. & Monod, J. Kinetics of the allosteric interactions of phosphofructokinase from *Escherichia coli*. *J. Mol. Biol.* **31**, 13-35 (1968).

5 Baldwin, S. A. & Perham, R. N. Novel kinetic and structural properties of the class-I D-fructose 1,6-bisphosphate aldolase from *Escherichia coli* (Crookes' strain). *Biochem. J.* **169**, 643-652 (1978).

6 Zgiby, S. M., Thomson, G. J., Qamar, S. & Berry, A. Exploring substrate binding and discrimination in fructose1, 6-bisphosphate and tagatose 1,6-bisphosphate aldolases. *Eur. J. Biochem.* **267**, 1858-1868 (2000).

7 Edgar, J. R. & Bell, R. M. Biosynthesis in *Escherichia coli* of sn-glycerol 3-phosphate, a precursor of phospholipid: Kinetic characterization of wild type and feedback-resistant forms of the biosynthetic sn-glycerol-3-phosphate dehydrogenase. *J. Biol. Chem.* **253**, 6354-6363 (1978).

8 Fifis, T. & Scopes, R. K. Purification of 3-phosphoglycerate kinase from diverse sources by affinity elution chromatography. *Biochem. J.* **175**, 311-319 (1978).

9 Young, T. A., Skordalakes, E. & Marqusee, S. Comparison of proteolytic susceptibility in phosphoglycerate kinases from yeast and *E. coli*: modulation of conformational ensembles without altering structure or stability. *J. Mol. Biol.* **368**, 1438-1447 (2007).

10 Schmidt, P. P., Travers, F. & Barman, T. Transient and equilibrium kinetic studies on yeast 3-phosphoglycerate kinase. Evidence that an intermediate containing 1,3-bisphosphoglycerate accumulates in the steady state. *Biochemistry* **34**, 824-832 (1995).

11 Fraser, H. I., Kvaratskhelia, M. & White, M. F. The two analogous phosphoglycerate mutases of *Escherichia coli*. *FEBS Lett.* **455**, 344-348 (1999).

12 Spring, T. G. & Wold, F. The purification and characterization of *Escherichia coli* enolase. *J. Biol. Chem.* **246**, 6797-6802 (1971).

13 Kuhnel, K. & Luisi, B. F. Crystal structure of the *Escherichia coli* RNA degradosome component enolase. *J. Mol. Biol.* **313**, 583-592 (2001).

14 Waygood, E. B., Mort, J. S. & Sanwal, B. D. The control of pyruvate kinase of *Escherichia coli:* Binding of substrate and allosteric effectors to the enzyme activated by fructose 1,6-bisphosphate. *Biochemistry* **15**, 277-282 (1976).

15 Kotlarz, D., Garreau, H. & Buc, H. Regulation of the amount and of the activity of phosphofructokinases and pyruvate kinases in *Escherichia coli*. *Biochim. Biophys. Acta* **381**, 257-268 (1975).

16 Waygood, E. B., Rayman, M. K. & Sanwal, B. D. The control of pyruvate kinases of *Escherichia coli.* II. Effectors and regulatory properties of the enzyme activated by ribose 5-phosphate. *Can. J. Biochem.* **53**, 444-454 (1975).
17 Waygood, E. B. & Sanwal, B. D. The control of pyruvate kinases of *Escherichia coli*. I. Physicochemical and regulatory properties of the enzyme activated by fructose 1,6-diphosphate. *J. Biol. Chem.* **249**, 265-274 (1974).
18 Tarmy, E. M. & Kaplan, N. O. Kinetics of *Escherichia coli* B D-lactate dehydrogenase and evidence for pyruvate-controlled change in conformation. *J. Biol. Chem.* **243**, 2587-2596 (1968).
19 Schomburg, I. *et al.* BRENDA: a resource for enzyme data and metabolic information. *Trends Biochem. Sci.* **27**, 54-56 (2002).
20 Bujara, M., Schümperli, M., Pellaux, R., Heinemann, M. & Panke, S. Optimization of a blueprint for *in vitro* glycolysis by metabolic real-time analysis. *Nat. Chem. Biol.* **7**, 271-277 (2011).
21 Baba, T. *et al.* Construction of Escherichia coli K-12 in-frame, single-gene knockout mutants: the Keio collection *Mol. Syst. Biol.* **2**, 2006.0008 (2006).
22 Annesley, T. M. Ion suppression in mass spectrometry. *Clin. Chem.* **49**, 1041-1044 (2003).
23 Wu, L. *et al.* Quantitative analysis of the microbial metabolome by isotope dilution mass spectrometry using uniformly 13C-labeled cell extracts as internal standards. *Anal. Biochem.* **336**, 164-171 (2005).
24 Flamholz, A., Noor, E., Bar-Even, A. & Milo, R. eQuilibrator-the biochemical thermodynamics calculator. *Nucleic Acids Res.* **40**, D770-D775 (2012).
25 Müller, C. L. & Sbalzarini, I. F. in *EvoApplications 2010, Part 1* (eds C. DiChic *et al.*) 432-441 (Springer-Verlag, 2010).
26 Goldberg, R. N. & Tewari, Y. B. Thermodynamics of enzyme-catalyzed reactions. 4. Lyases. *J. Phys. Chem. Ref. Data* **24**, 1669-1698 (1995).
27 Bakker, B. M., Michels, P. A. M., Opperdoes, F. R. & Westerhoff, H. V. Glycolysis in bloodstream form *Trypanosoma brucei* can be understood in terms of the kinetics of the glycolytic enzymes. *J. Biol. Chem.* **272**, 3207-3215 (1997).
28 Chassagnole, C., Noisommit-Rizzi, N., Schmid, J. W., Mauch, K. & Reuss, M. Dynamic modeling of the central carbon metabolism of *Escherichia coli*. *Biotechnol. Bioeng.* **79**, 53-73 (2002).
29 Baldwin, S. A., Perham, R. N. & Stribling, D. Purification and characterization of the class-II D-fructose 1,6-bisphosphate aldolase from *Escherichia coli* (Crookes' strain). *Biochem. J.* **169**, 633-641 (1978).
